# 3′ Untranslated Regions Mediate Transcriptional Interference between Convergent Genes Both Locally and Ectopically in *Saccharomyces cerevisiae*

**DOI:** 10.1371/journal.pgen.1004021

**Published:** 2014-01-23

**Authors:** Luwen Wang, Ning Jiang, Lin Wang, Ou Fang, Lindsey J. Leach, Xiaohua Hu, Zewei Luo

**Affiliations:** 1 Laboratory of Population & Quantitative Genetics, Institute of Genetics and Biostatistics, SKLG, School of Life Sciences, Fudan University, Shanghai, China; 2 School of Biosciences, The University of Birmingham, Birmingham, United Kingdom; University of Michigan, United States of America

## Abstract

Paired sense and antisense (S/AS) genes located in *cis* represent a structural feature common to the genomes of both prokaryotes and eukaryotes, and produce partially complementary transcripts. We used published genome and transcriptome sequence data and found that over 20% of genes (645 pairs) in the budding yeast *Saccharomyces cerevisiae* genome are arranged in convergent pairs with overlapping 3′-UTRs. Using published microarray transcriptome data from the standard laboratory strain of *S. cerevisiae*, our analysis revealed that expression levels of convergent pairs are significantly negatively correlated across a broad range of environments. This implies an important role for convergent genes in the regulation of gene expression, which may compensate for the absence of RNA-dependent mechanisms such as micro RNAs in budding yeast. We selected four representative convergent gene pairs and used expression assays in wild type yeast and its genetically modified strains to explore the underlying patterns of gene expression. Results showed that convergent genes are reciprocally regulated in yeast populations and in single cells, whereby an increase in expression of one gene produces a decrease in the expression of the other, and vice-versa. Time course analysis of the cell cycle illustrated the functional significance of this relationship for the three pairs with relevant functional roles. Furthermore, a series of genetic modifications revealed that the 3′-UTR sequence plays an essential causal role in mediating transcriptional interference, which requires neither the sequence of the open reading frame nor the translation of fully functional proteins. More importantly, transcriptional interference persisted even when one of the convergent genes was expressed ectopically (in *trans*) and therefore does not depend on the *cis* arrangement of convergent genes; we conclude that the mechanism of transcriptional interference cannot be explained by the transcriptional collision model, which postulates a clash between simultaneous transcriptional processes occurring on opposite DNA strands.

## Author Summary

In the compact genome of the budding yeast *Saccharomyces cerevisiae*, genes are frequently organized into convergent pairs that are transcribed from opposing DNA strands in opposite directions and have overlapping 3′-UTRs. Here we explore the negative correlation in expression levels between convergent genes using a set of 645 convergent pairs in the yeast genome, identified from published genomic and transcriptomic sequence data and accounting for ∼20% of total yeast genes. Analysis of published microarray experiments confirmed that the negative correlation in expression between convergent genes occurs across a broad range of growth conditions. This implies that such transcriptional interference is an important means of regulating gene expression in yeast, especially in the absence of other eukaryotic RNA-dependent mechanisms such as micro RNAs. We focused on profiling the expression of four pairs of convergent genes in wild type yeast and its genetically modified strains, to explore the causes and mechanisms of transcriptional interference. We demonstrate that the 3′-UTR sequence alone plays the essential and causal role in interference between convergent genes. Intriguingly, transcriptional interference occurs even when one of the convergent genes is expressed from elsewhere in the genome (in *trans*), raising new questions about how transcriptional interference operates.

## Introduction

Sense and anti-sense transcripts (S/AS) are simply pairs of RNAs (either protein-coding or non-protein-coding) containing sequences that are at least partially complementary to each other. S/AS gene pairs can be transcribed in *cis* from opposing DNA strands at the same genomic locus [1]. In recent years, genome sequencing projects have revealed the frequent presence of antisense transcripts in cells, even in one of the smallest self-replicating organisms, *Mycoplasma pneumoniae* [2]. In particular, S/AS pairs located in *cis* represent a common structural feature in the genomes of both prokaryotes and eukaryotes, including mammals (human, mouse, rat, cow), birds (chicken), lower vertebrates (zebrafish), invertebrates (*Caenorhabditis elegans, Drosophila melanogaster*), plants (*Arabidopsis thaliana*, rice), and yeast [3]–[6]. According to their transcriptional orientation and extent of sequence overlap, S/AS genes can be classified into three major groups: (1) convergent gene pairs, overlapping at their 3′ ends; (2) divergent gene pairs, overlapping at their 5′ ends; and (3) consistent gene pairs, overlapping and transcribed in the same direction. The genomic distribution of these different types of gene pair is species-specific. For example, convergent gene pairs are prevalent in *Drosophila* and *C. elegans*, but are rare in human and mouse genomes [3].

The structural organization of S/AS gene pairs confers a significant mechanism for regulating gene expression levels. For example, global analysis of the mammalian transcriptome showed that a large proportion of the genome can produce transcripts from both strands and revealed anti-regulation of S/AS pairs [7]. In particular, experimental perturbation of an antisense RNA was shown to alter the expression of the sense mRNA in *cis*. In both budding and fission yeasts, transcriptional interference has been observed between sense and antisense transcripts of several convergent genes arranged in *cis* or *trans* [8]–[12]. This involves one transcriptional process exerting a direct negative impact on a second transcriptional process. Such interference plays important functional roles, for example in the entry of yeast cells into meiosis by regulating the expression of the *IME4* gene [9].

Antisense transcripts are mature RNA species (polyadenylated at their 3′ ends) and contribute a mechanism of transcription interference that is distinct from the RNAi-mediated regulation of gene expression that is present in most eukaryotes, but absent in *Saccharomyces cerevisiae*. For example, antisense transcripts involved in RNAi are often encoded elsewhere in the genome from the target gene, and require further processing into shorter, functional sequences by the RNAi machinery. The most obvious explanation for transcriptional interference between convergent S/AS gene pairs is given by the collision model [13]; in this model, RNA synthesis from one DNA strand clashes with transcription from the other strand, and so active antisense transcription would suppress sense RNA transcription [14], [15]. Though this model is supported by atomic force microscopy data in *E. coli* [16], its role in transcriptional interference in budding yeast has not been thoroughly assessed.

In this work, we have exploited the availability of genome-wide transcriptome data from the budding yeast *S. cerevisiae* to explore the significance of transcriptional interference between convergent gene pairs on a global scale. Using a comprehensive set of S/AS pairs with overlapping 3′-UTRs, we have demonstrated that transcriptional interference is common to more than 600 gene pairs, which account for ∼20% ORFs in the *S. cerevisiae* genome, across a broad range of growth conditions. For a more detailed understanding of the underlying mechanisms, we focus on four such representative gene pairs and show that transcriptional interference is dependent only on the 3′-UTR sequence. Moreover, its occurrence is not restricted to the arrangement of the gene pairs in *cis*, but can also occur when the partner genes are re-located apart (in *trans*). We have illustrated the functional importance of this mode of gene regulation during the yeast cell cycle and its role in the phenotypic response of yeast cells to environmental stress.

## Results

### Genome-wide transcriptional interference of convergent genes in the yeast genome

Based on genome-wide transcriptome [17] and genome sequence data, we identified 645 convergent gene (ORF) pairs with overlapping 3′-UTRs, accounting for ∼20% of total genes in the yeast genome (see materials and methods, **Table S1**). We compared the convergent ORF pairs predicted from the mRNA-Seq data with those from both nascent RNA sequencing (NET-Seq) data [18] and strand-specific RNA sequencing (ssRNA-Seq) data [19]. We found that among the 645 convergent pairs, 531 (82.3%) and 329 (51.0%) were detected as convergent pairs with overlapping 3′-UTRs from the NET-Seq and ssRNA-Seq datasets respectively. For 15.5% and 33.0% of the 645 convergent pairs, at least one of the two genes in the pair was unexpressed, in the NET-Seq and ssRNA-Seq datasets respectively (Figure S1). Additionally, there are only 12M sequencing reads in the ssRNA-Seq dataset, whilst the mRNA-Seq and NET-Seq datasets contain 29M and 69M reads respectively. This may, at least partly, explain the lower proportion of the convergent pairs confirmed in the ssRNA-Seq data compared to the NET-Seq data.

Based on the mRNA-Seq and genomic sequencing data, we observed a highly significant negative correlation between 3′-UTR length and gene expression level of the corresponding ORF (*r* = −0.089, *P*<10^−4^), suggesting an important role for the 3′-UTR in the regulation of transcription. However, there was no clear relationship between 5′-UTR length and gene expression level. To assess the occurrence of transcriptional interference between convergent partner genes with overlapping 3′-UTRs, we extracted mRNA expression values for these 645 gene pairs from Affymetrix microarray experiments based on the test strain BY4741 under seven environmental stress conditions (**Table S3**). The expression of convergent ORFs showed a consistent and highly significant negative correlation (−0.159≤*r≤*−0.124; 10^−4^≤P≤0.002) across all seven conditions (**Figure S2**). The significant negative correlation was also observed in both the NET-Seq (*r* = −0.09, *P*<0.05) and ssRNA-Seq data (*r* = −0.121, *P*<0.05). We infer that transcriptional interference between convergent gene pairs with overlapping 3′-UTRs is a widespread phenomenon in the yeast genome.

### Transcriptional interference between convergent genes located in *cis* and in *trans*

**Figure 1:**
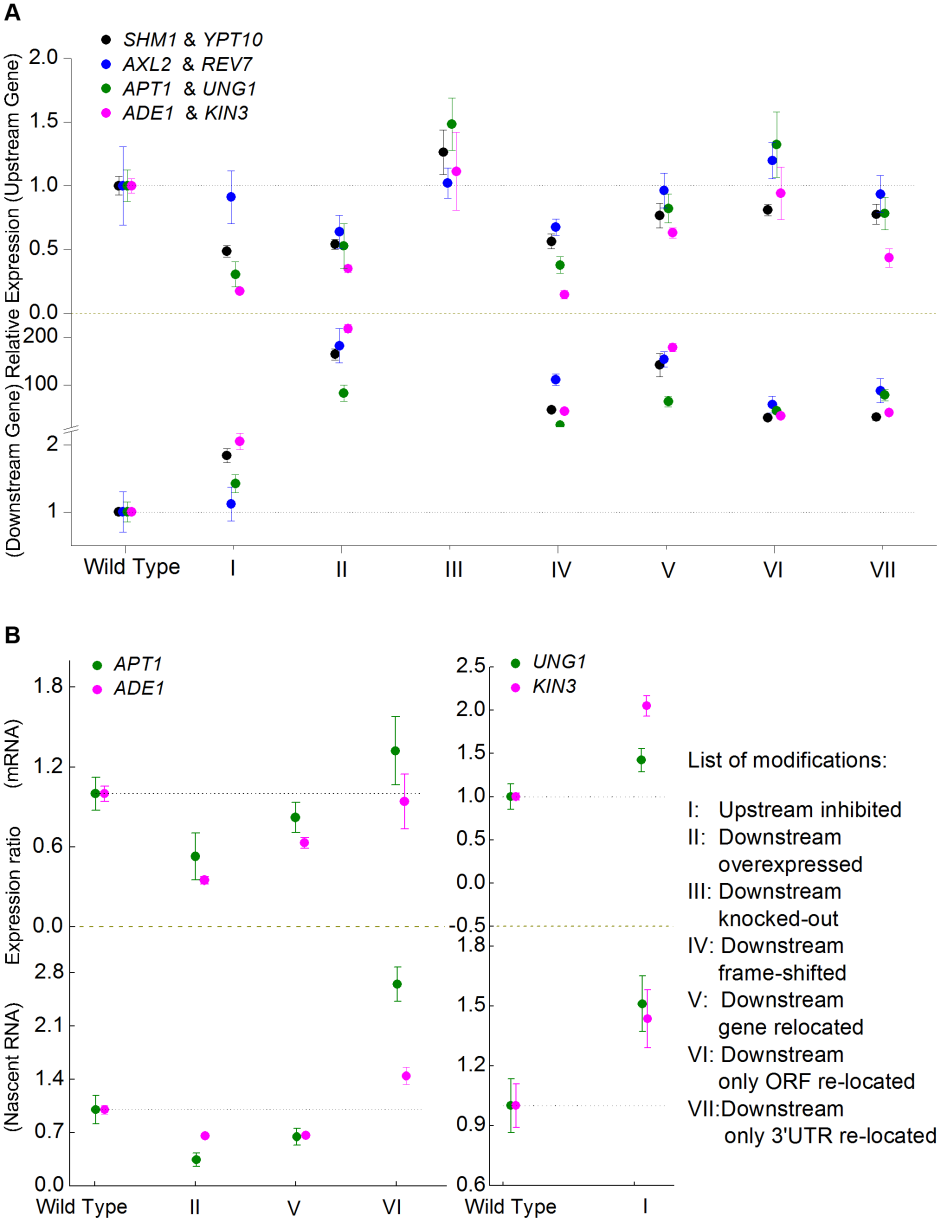
RT-PCR expression assay of convergent gene pairs located in *cis* or in *trans*. (**A**) Expression levels of four pairs of convergent genes with overlapping 3′-UTRs in wild type yeast, or when the upstream (group I) or the downstream (groups II–VII) genes were modified by: (I) inhibition of gene expression; (II) over-expression; (III) gene knock-out; (IV) frame-shift of the ORF; (V) re-location of the entire ORF and 3′-UTR; (VI) re-location of only the ORF; and (VII) re-location of only the 3′-UTR. The mean and standard deviation of expression level for three replicates are given in relative units compared to the wild type genes, which are assigned a value of 1.0. (**B**) Expression levels of nascent and mature messenger RNA for one of two convergent genes when its convergent partner's expression was altered.

To further understand the transcriptional relationship between genes with overlapping 3′-UTRs, we selected four convergent gene pairs from the complete set of 645 pairs for more detailed study (**Table 1**). The 3′-UTR boundaries and overlapping regions were confirmed by 3′-RACE sequencing. In addition, these four pairs were also confirmed as convergent genes with overlapping 3′-UTRs in the NET-Seq and ssRNA-Seq datasets (**Figure S3**). We profiled gene expression of the convergent pairs in the wild type strain YL1C^WT^, a laboratory haploid strain which we have described previously [20], and in a series of seven derived genetically engineered strains (**Table 2**), as illustrated in **Figure 1A**. When expression of upstream genes (*SHM1*, *AXL2*, *APT1* and *ADE1*) was inhibited (group I), the expression of the downstream partner genes (*YPT10*, *REV7*, *UNG1* and *KIN3*) was up-regulated in comparison to the wild type control. Correspondingly, when the downstream genes were over-expressed, expression of the upstream partner genes was clearly repressed (group II). In the extreme situation where expression of the downstream genes was completely silenced (group III), expression of the upstream genes increased to a variable extent compared to the wild type level. Convergent overlapping gene pairs therefore exhibit a pattern whereby a change in the expression of either partner gene leads to the expression of the other gene changing in the opposite direction, a pattern we refer to as ‘anti-regulation’ hereafter.

**Table 1:** Four convergent pairs of *S. cerevisiae* ORFs with overlapping 3′-UTRs.

ORF pair	3′-UTR length	Length of sequence overlap	Biological process
AXL2 REV7	127 bp 178 bp	164 bp	Axial cellular bud site selection in cell-cycle Error-free/error-prone translesion synthesis
APT1 UNG1	211 bp 180 bp	157 bp	Adenine biosynthesis DNA repair, regulated by cell-cycle
ADE1 KIN3	98 bp 206 bp	206 bp	Purine nucleotide biosynthetic process Protein kinase, mitotic spindle orientation
SHM1 YPT10	53 bp 9 bp	29 bp	Serine family amino acid biosynthesis Golgi organization

The upstream (downstream) gene is shown in row 1 (2).

**Table 2:** Summary of genetically modified strains of the standard wild type yeast YL1C.

Group	Modification targeted	Strains	Modifications made
I	Upstream gene inhibited	S-Y1	*shm1* promoter :: T_TEF_ (terminator inserted upstream of *SHM1^ORF^*)
		X-R1	*axl2* promoter :: T_TEF_ (terminator inserted upstream of *AXL2^ORF^*)
		P-U1	*apt1* promoter :: T_TEF_ (terminator inserted upstream of *APT1^ORF^*)
		D-K1	*ade1* promoter :: T_TEF_ (terminator inserted upstream of *ADE1^ORF^*)
II	Downstream gene over- expressed	S-Y2	P_ADH_-*YPT10^wt^* (P_ADH_ inserted upstream of *YPT10^ORF^*)
		X-R2	P_ADH_-*REV7^wt^* (P_ADH_ inserted upstream of *REV7^ORF^*)
		P-U2	P_ADH_-*UNG1^wt^* (P_ADH_ inserted upstream of *UNG1^ORF^*)
		D-K2	P_ADH_-*KIN3^wt^* (P_ADH_ inserted upstream of *KIN3^ORF^*)
III	Downstream gene knocked-out	S-Y3	*ypt10^ORF^* :: *NAT1* (*YPT10^ORF^* knocked out)
		X-R3	*rev7^ORF^* :: *NAT1* (*REV7^ORF^* knocked out)
		P-U3	*ung1^ORF^* :: *NAT1* (*UNG1^ORF^* knocked out)
		D-K3	*kin3^ORF^* :: *NAT1* (*KIN3^ORF^* knocked out)
IV	Downstream gene frame shifted	S-Y4	P_ADH_-ypt*10^mut^* (Frameshift gene *ypt10* over-expressed by P_ADH_)
		X-R4	P_ADH_-*rev7^mut^* (Frameshift gene *rev7* over-expressed by P_ADH_)
		P-U4	P_ADH_-*ung1^mut^* (Frameshift gene *ung1* over-expressed by P_ADH_)
		D-K4	P_ADH_-*kin3^mut^* (Frameshift gene *kin3* over-expressed by P_ADH_)
V	Downstream gene (ORF+3′- UTR) ectopically expressed	S-Y5	*ho* ::*YPT10^ORF+3′-UTR^* (*YPT10^ORF^* transferred to *HO* locus with its 3′-UTR)
		X-R5	*ho*::*REV7^ORF+3′-UTR^* (*REV7^ORF^* transferred to *HO* locus with its 3′-UTR)
		P-U5	*ho*::*UNG1^ORF+3′-UTR^* (*UNG1^ORF^* transferred to *HO* locus with its 3′-UTR)
		D-K5	*ho*::*KIN3^ORF+3′-UTR^* (*KIN3^ORF^* transferred to *HO* locus with its 3′-UTR)
VI	Downstream gene (ORF only) ectopically expressed	S-Y6	*ho* ::*YPT10^ORF^* (*YPT10^ORF^* transferred to *HO* locus without its 3′-UTR)
		X-R6	*ho*:: *REV7^ORF^* (*REV7^ORF^* transferred to *HO* locus without its 3′-UTR)
		P-U6	*ho*::*UNG1^ORF^* (*UNG1^ORF^* transferred to *HO* locus without its 3′-UTR)
		D-K6	*ho*::*KIN3^ORF^* (*KIN3^ORF^* transferred to *HO* locus without its 3′-UTR)
VII	Downstream gene (3′-UTR only) ectopically expressed	S-Y7	*ho* :: *Sh ble*+3′-UTR of *ypt10* (fusion gene transferred to *HO* locus)
		X-R7	*ho*:: *Sh ble*+3′-UTR of *rev7* (fusion gene transferred to *HO* locus)
		P-U7	*ho*:: *Sh ble*+3′-UTR of *ung1* (fusion gene transferred to *HO* locus)
		D-K7	*ho*:: *Sh ble*+3′-UTR of *kin3* (fusion gene transferred to *HO* locus)
VIII	Terminator changed	P-U8	T_ADH_-*APT1^wt^* & T_AOX1_-*UNG1^wt^* (Changing intergenic region with independent terminators)
		D-K8	T_ADH_-*ADE1^wt^* & T_AOX1_-*KIN3^wt^* (Changing intergenic region with independent terminators)
IX	Terminator changed and upstream gene inhibited	P-U9	*apt1* promoter :: T_TEF_ (terminator inserted upstream of *APT1^ORF^* in P-U8)
		D-K9	*ade1* promoter :: T_TEF_ (terminator inserted upstream of *ADE1^ORF^* in D-K8)
X	Terminator changed and downstream gene over- expressed	P-U10	P_ADH_-*UNG1^wt^* (P_ADH_ inserted upstream of *UNG1^ORF^* in P-U8)
		D-K10	P_ADH_-*KIN3^wt^* (P_ADH_ inserted upstream of *KIN3^ORF^* in D-K8)

P_ADH_ is a strong constitutive promoter.

To explore the relationship between transcriptional interference and the sequence of the ORF, and thus of the protein encoded, we created frame-shift mutations in the coding sequence of the downstream genes. These mutations were designed to generate premature termination in translation, which, while not changing the normal transcription of the genes, would mean that the transcripts generated would no longer be translated into normally functioning proteins. However, anti-regulation (transcriptional interference) was still observed in the presence of such mutations (group IV). Expression of the frame-shifted downstream genes was increased relative to that of the wild types, while at the same time, expression of the upstream genes was correspondingly decreased. This shows that over-expressing the mutated downstream genes leads to significantly altered expression of their upstream convergent partners, and thus excludes the possibility that anti-regulation of gene partners in these four convergent pairs is dependent on functional interactions between their encoded proteins.

Having established the transcriptional interference between the four pairs of convergent genes with overlapping 3′-UTRs, we then created a series of genetically modified strains to test if the interference could be maintained in *trans*. These modifications included translocation of either the complete (ORF and 3′-UTR) or partial (ORF or 3′-UTR only) sequence of the downstream genes to the *HO* locus and ectopic expression stimulated by the constitutive promoter P_ADH_. Ectopic over-expression of the complete downstream genes (group V) or only their 3′-UTRs (group VII) significantly repressed the expression of the upstream genes. In contrast, over-expression of the ORF alone did not lead to an obvious decrease in expression of the upstream genes (group VI). Firstly, this shows that the 3′-UTR (rather than the ORF) plays a key role in anti-regulation between the partner genes of convergent gene pairs. Secondly, the 3′-UTRs can mediate anti-regulation of expression of the partner genes in *trans*, *i.e.* when the partner genes are located apart, and therefore does not depend on their native arrangement in *cis*. It should be noted that the range of variation in relative expression of downstream genes is large in comparison to that of the corresponding upstream genes in groups II, IV, V, VI and VII. This reflects the fact that we designed the experiments to overexpress the downstream genes.

We compared the transcriptional interference at both mature RNA and nascent RNA levels for two of the four convergent gene pairs. **Figure 1B** illustrates expression of one gene when expression of its convergent partner was altered for two convergent gene pairs at nascent RNA (lower panel) and mature mRNA (upper panel) levels. It is clear that expression of one of the convergent genes markedly responds to altered expression of its convergent partner at both nascent and mature RNA levels. This excludes the possibility that the anti-regulation of expression between convergent gene pairs occurs during the RNA maturation process.

### Transcriptional interference between convergent genes is reflected in protein abundance

**Figure 2:**
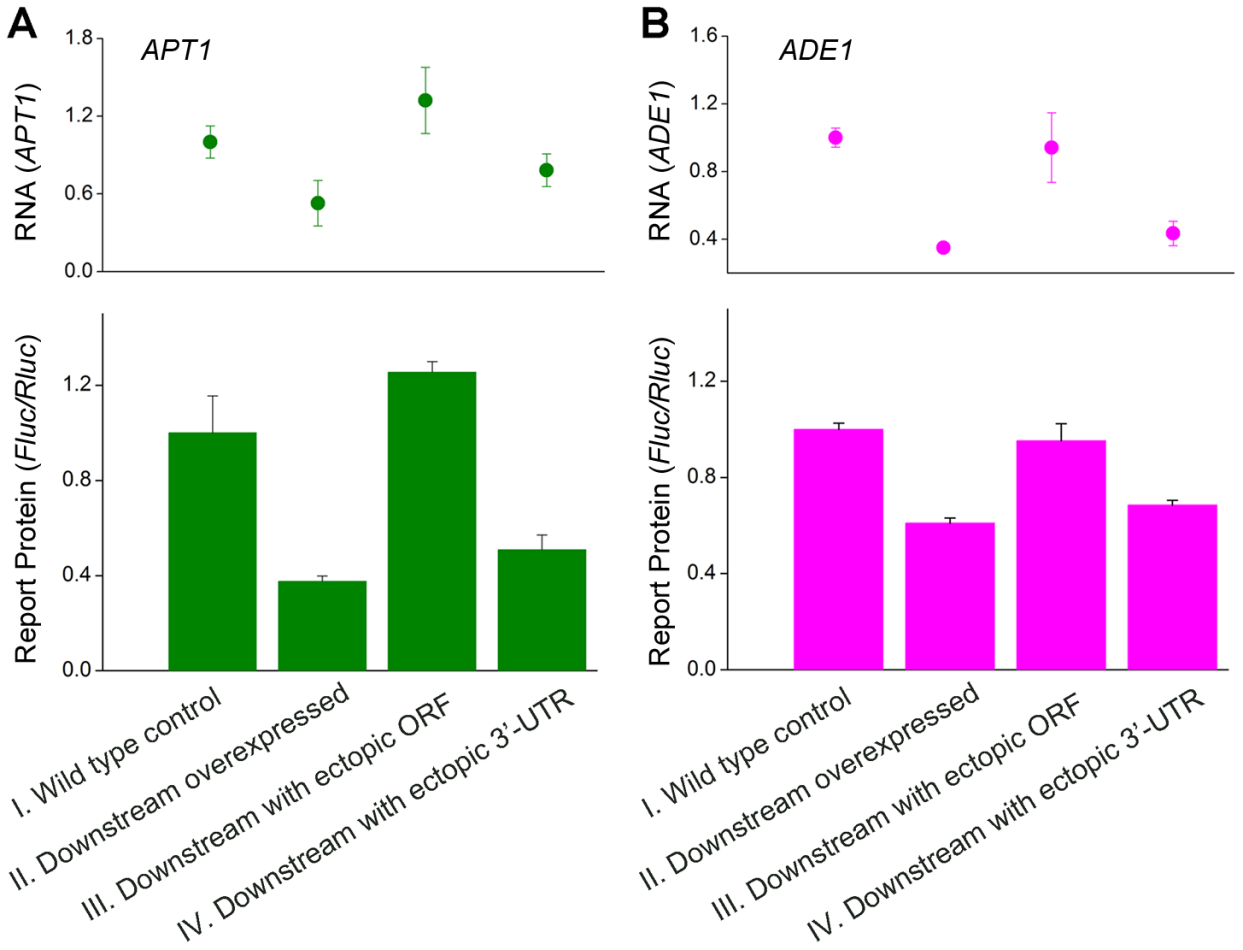
Dual-luciferase reporter assay of protein levels for two convergent gene pairs. Dual-luciferase reporter assay for the expression of the upstream genes of two pairs of convergent genes with overlapping 3′-UTRs. The reporter protein replaced the ORF of each upstream gene (**A** *APT1* or **B** *ADE1*), while the downstream genes were modified: (I) wild type control with no modification; (II) over-expression of the entire gene; (III) ectopic over-expression of the ORF alone; and (IV) ectopic over-expression of the 3′-UTR alone. Mean and standard deviation based on three replicates are shown for mRNA expression level (upper panel) and protein abundance (lower panel), measured in relative units compared to the wild type control, which was assigned a value of 1.0.

We next sought to determine whether the observed anti-regulation in expression between convergent gene pairs with overlapping 3′-UTRs could also be observed at the level of protein abundance. We replaced the ORFs of the upstream partners for two of the four gene pairs (*APT1*/*UNG1* and *ADE1*/*KIN3*) with the reporter gene *Fluc*, and quantified expression of the reporter protein in the wild type and modified backgrounds. Over-expression of either the downstream genes in *situ*, or of their 3′-UTRs ectopically, caused significant reduction in levels of the reporter protein (**Figure 2**). However, ectopic over-expression of only the ORF did not alter levels of the reporter protein. Therefore interference between convergent genes with overlapping 3′-UTRs affects both the expression of gene transcripts and protein levels. These effects are dependent on the 3′-UTR sequence and can be observed in *trans* as well as in *cis*.

**Figure 3:**
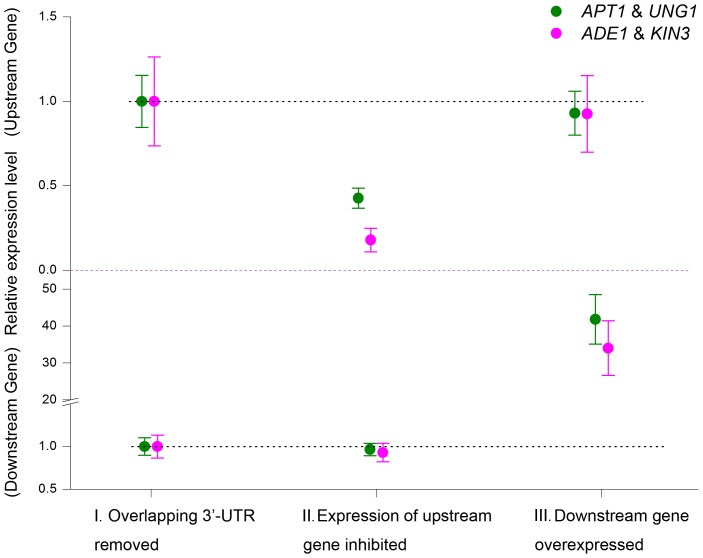
Loss of anti-regulation in the absence of overlapping 3′-UTRs. Expression levels of the upstream and downstream genes of two convergent gene pairs with their overlapping 3′-UTRs removed. Mean and standard deviation based on three replicates are given for the expression levels: (I) for a baseline control, assigned a value of 1.0; (II) with inhibition of the upstream gene; or (III) with over-expression of the downstream gene.

To further confirm the causal role of the overlapping 3′-UTRs in the transcriptional interference between convergent gene pairs, we removed the overlapping 3′-UTRs of two convergent pairs (*APT1*/*UNG1* and *ADE1*/*KIN3*) and measured their expression responses (**Figure 3**, group I). Inhibition of expression of the upstream gene did not result in up-regulation of the corresponding downstream partner gene (group II). Similarly, over-expression of the downstream gene did not lead to repression of the upstream partner gene (group III). These results reveal that the 3′-UTR plays a crucial, causal role in the anti-regulation of expression between convergent gene pairs with overlapping 3′-UTRs.

### Convergent genes reach peak expression values at different times in the cell-cycle

**Figure 4:**
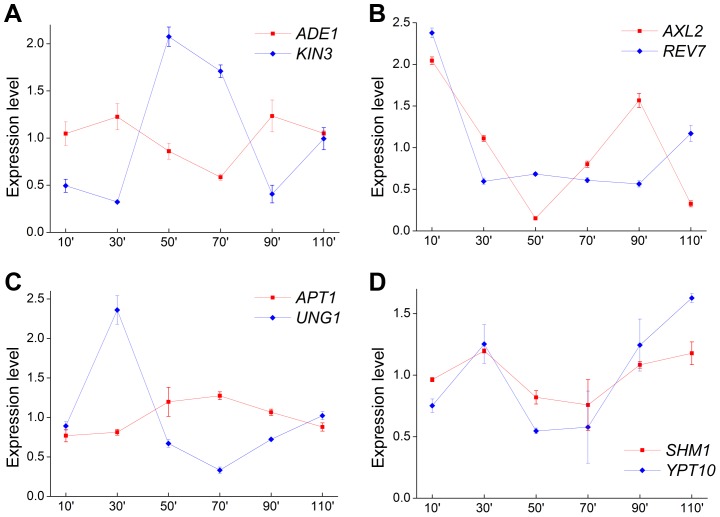
Expression patterns of four convergent gene pairs during the yeast cell cycle. Expression of four pairs of yeast convergent genes with overlapping 3′-UTRs in cells from the wild type strain YL1A, the a-mating strain of YL1C (**A** *ADE1/KIN3*, **B** *AXL2/REV7*, **C** *APT1/UNG1* and **D** *SHM1/YPT10*). Gene expression was assessed 10 minutes from the start of the cell-cycle and at 20 minutes intervals thereafter.

Three of the four convergent gene pairs involve a biosynthesis pathway gene and a cell-cycle dependent gene (**Table 1**), providing an opportunity to investigate the dynamic change in expression of the convergent gene pairs over the course of the cell cycle. We profiled the expression of each pair over the 100 minute time span of the cell cycle and found a consistent pattern whereby one ORF reaches its expression peak, while its convergent partner is suppressed (**Figure 4A–C**). The fourth gene pair involves a biosynthesis gene (*SHM1*) and a GTP-binding protein (*YPT10*). As expected, this pair did not follow the same pattern (**Figure 4D**). This demonstrates the functional importance of transcriptional interference between convergent gene pairs in the cell cycle of budding yeast.

**Figure 5:**
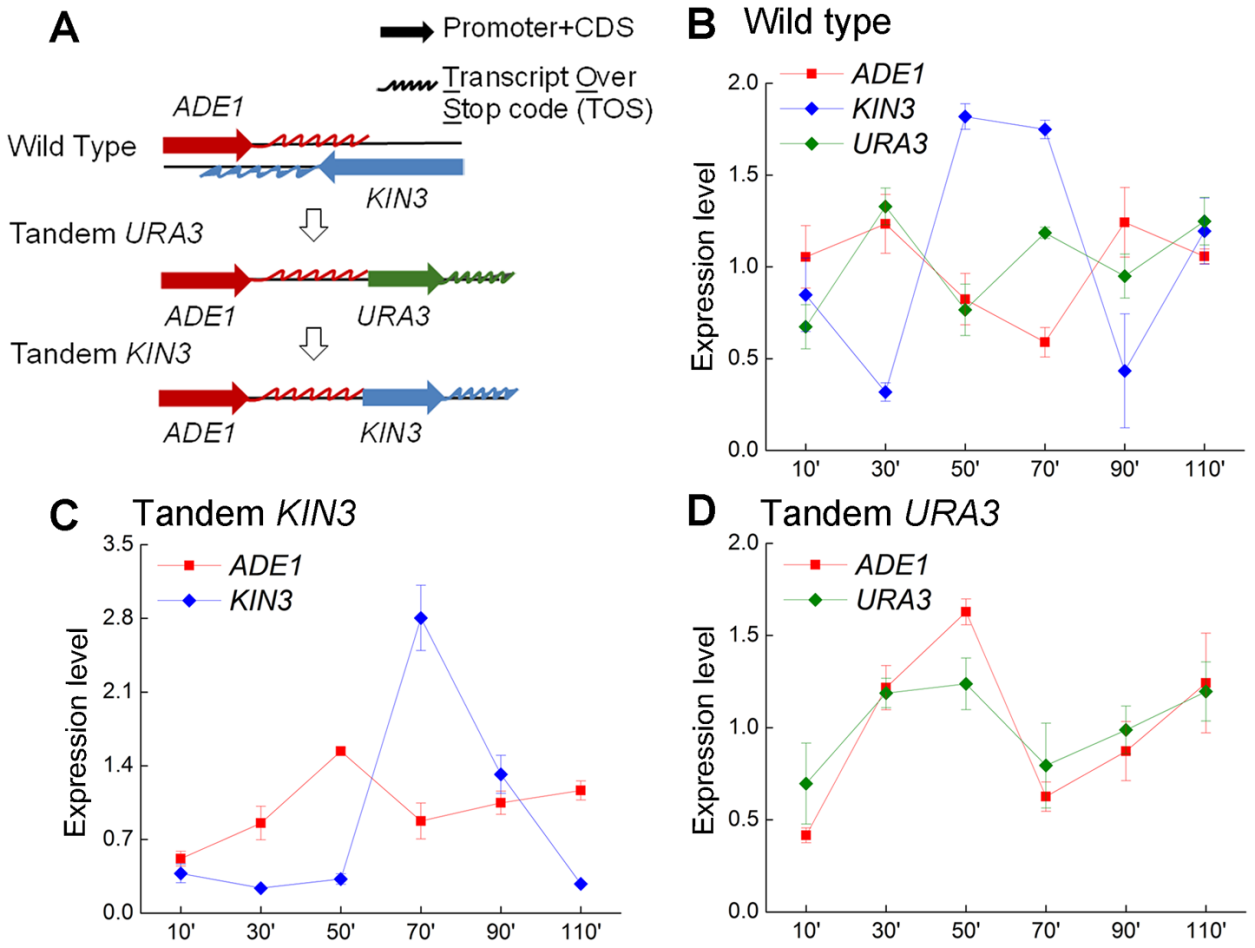
Cell-cycle expression of the gene pair *ADE1* and *KIN3*, in convergent and tandem orientation. (**A**) Three strains tested: (I) wild type, in which *ADE1* and *KIN3* were in convergent orientation; (II) tandem *URA3*, in which *KIN3* was replaced with *URA3*, and *ADE1* and *URA3* were in tandem orientation; and (III) tandem *KIN3*, in which *ADE1* and *KIN3* were in tandem orientation. (**B**) Cell cycle expression of the three genes *ADE1*, *KIN3* and *URA3* in the wild type strain. (**C**) Cell cycle expression pattern of the genes *ADE1* and *KIN3* in tandem orientation. (**D**) Expression of the genes *ADE1* and *URA3* in the tandem *URA3* strain.

We explored the cell-cycle expression pattern of one of the four gene pairs with overlapping 3′-UTRs (*ADE1*/*KIN3*) when its orientation was switched from ‘convergent’ to ‘tandem’. We created two genetically modified strains through homologous recombination, the tandem *KIN3* and the tandem *URA3*, as illustrated in **Figure 5A**. The modifications were confirmed by sequencing (the sequence data is listed in **Table S5**). **Figure 5B** illustrates the cell cycle expression of the three genes *ADE1*, *KIN3* and *URA3* in the wild type strain. It is clear that cell-cycle expression of the tested convergent gene pair was repeatedly confirmed in this independent assay, showing the expression pattern of one-rising and the other falling, as shown in **Figure 4A**. When the convergent pair was converted into ‘tandem’ orientation, the cell-cycle expression patterns of the convergent pair changed in comparison to that of the genes in wild type background, but the pattern of one rising and the other falling remained (**Figure 5C**). Moreover, we observed the expression pattern of *ADE1* was also markedly altered when its downstream partner gene was replaced with a tandemly oriented gene, *URA3* (**Figure 5D**). These observations strongly support the anti-regulation in the cell cycle expression of the convergent pair, which is independent of genomic orientation of the convergent genes. A natural question arises whether each gene in the convergent gene pairs tested is expressed in the same cell or instead is expressed mainly in different cells in the cell population. To answer this question we profiled expression of the convergent pair, *KIN3* and *ADE1*, in single cells across different stages of the cell cycle (**Figure S4**). It shows a highly significantly negative correlation in expression between the convergent genes (Pearson's correlation coefficient *r* = −0.38, *P*<0.01), indicating that anti-regulation between convergent genes is also observed within individual cells.

### Phenotypic response to environmental change mediated by the 3′-UTRs of convergent genes

**Figure 6:**
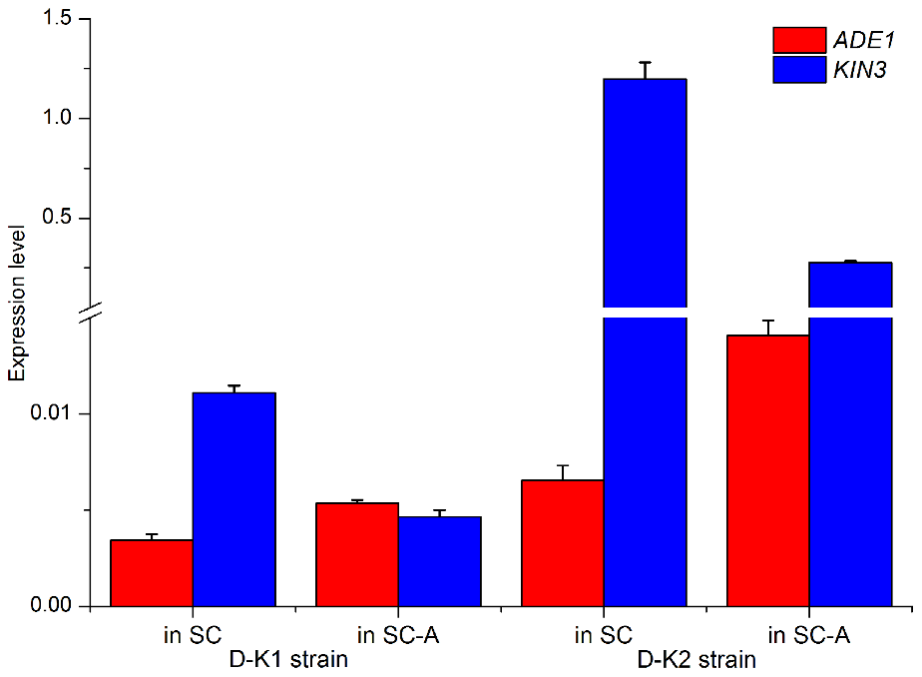
Anti-regulation of a convergent pair in different nutritional environments. Expression of the convergent gene pair (*ADE1* and *KIN3*) with overlapping 3′-UTRs in two yeast strains cultured in complete (SC) or adenine barren (SC-A) medium. Strain D-K1 has *ADE1* inhibited, while strain D-K2 has *KIN3* over-expressed.

It is well established that gene expression in the budding yeast *Saccharomyces cerevisiae* changes in response to nutrient availability [8], [21]. For example, *ADE1* expression is responsive to adenine availability in the culture medium; when yeast cells are cultured in adenine rich (synthetic complete, SC) medium, the expression of *ADE1* is suppressed, while its expression is stimulated in an adenine barren medium (SC-A) [22]. We examined expression of the convergent pair *ADE1*/*KIN3* in the modified strains D-K1 and D-K2 (**Table 2**), cultured in either adenine rich or barren medium. In both strains, *ADE1* expression was suppressed in the SC medium, but enhanced in the SC-A medium. Conversely, *KIN3* expression was increased in the SC medium and decreased in SC-A, even in the presence of the strong prompter P_ADH_ in the test strain D-K2 (**Figure 6**). These observations indicate that alterations in gene expression in response to nutrient availability can be achieved through anti-regulation of expression between partners of a convergent gene pair, mediated by their 3′-UTR.

**Figure 7:**
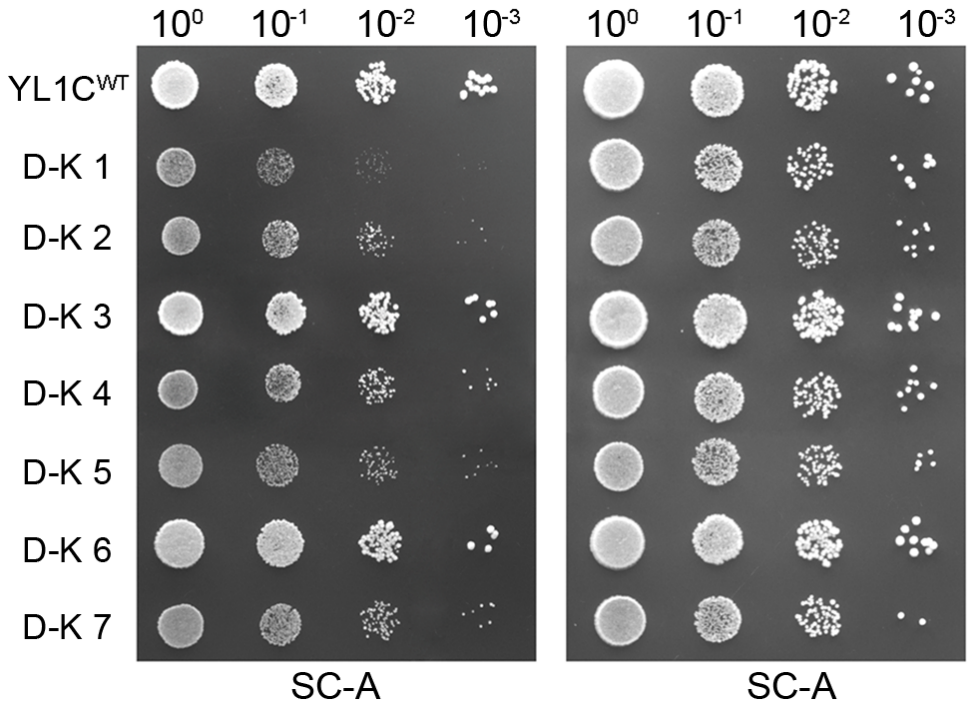
Phenotypic effect of anti-regulation between a convergent pair in a nutrient-limited environment. Growth phenotype of the wild type strain (YL1C^WT^) and seven strains (D-K1 to D-K7; see **Table 2**) genetically modified for the convergent gene pair *ADE1* and *KIN3*. Strains were cultured in complete (SC) or adenine barren (SC-A) medium.

We further tested the role of anti-regulation between convergent genes using a growth assay of the wild type strain (YL1C^WT^) and seven strains (D-K1 to D-K7, **Table 2**) genetically modified for the convergent gene pair *ADE1* and *KIN3*. In an adenine rich (SC) medium, the growth phenotype of all the strains largely reflects the change in the inoculation concentration (**Figure 7**, right panel). In the adenine-barren medium (SC-A), growth phenotype of the wild type strain is comparable to that in the rich medium, given that the *ADE1* gene is expressed at normal levels, as shown previously [22]. Growth of the strain with the *KIN3* gene knocked out (D-K3) is comparable to that of the wild type strain, agreeing with the fact that expression level of the *ADE1* gene is comparable between these two strains (group III, **Figure 1A**). However, growth is clearly repressed in the SC-A medium for strains in which *ADE1* expression was suppressed, either directly (D-K1) or indirectly by over-expression of its convergent partner gene *KIN3*, *in situ* (D-K2 and D-K4) or ectopically (D-K5). Ectopic over-expression of the *KIN3* 3′-UTR alone (D-K7) clearly repressed growth in comparison to the wild type, but no repression was observed with ectopic over-expression of the *KIN3* ORF alone (D-K6). Combining this result with our earlier observations suggests that ectopic over-expression of either the *KIN3* 3′-UTR or of the entire gene suppressed the expression of its convergent partner gene *ADE1*, which in turn, led to suppressed growth in the nutrient limited medium. We conclude that the convergent organization of genes with overlapping 3′-UTRs in the yeast genome constitutes an effective mechanism for regulating gene expression and ultimately for controlling cell growth.

## Discussion

The mechanisms of transcriptional regulation are highly conserved in eukaryotic species [23]. However, the yeast genome is distinct in several ways [24]. First, by the complete absence of RNA-dependent regulatory systems (including miRNAs and siRNAs) present in higher eukaryotes. Second, by its highly compact structure, for example, over 20% of ORFs in the yeast genome are arranged in *cis* as sense and antisense gene pairs (S/AS) that overlap and are transcribed from opposing DNA strands. To explore the expression pattern of genes arranged in this way, we analyzed a complete set of over 600 convergent gene pairs with overlapping 3′-UTRs. The convergent genes showed a significant and consistent pattern of negative correlation in expression across a broad range of growth conditions, though this negative correlation could only explain a limited fraction of total gene expression variability. The mechanism underlying this widespread transcriptional interference was explored in detail for four representative pairs of convergent genes.

We analyzed the expression patterns of the four convergent gene pairs in wild type yeast and its derived strains with various genetic modifications. The data revealed that convergent genes regulate each other's expression such that an increase in expression of either gene produces a decrease in expression of the other. We showed that this pattern of ‘anti-regulation,’ or transcriptional interference occurs in single cells and does not require interaction between the proteins encoded by the convergent genes, since the effects persisted when the downstream gene was mutated so that a fully functional protein could not be made. Furthermore, we demonstrate that transcriptional interference between convergent genes is reflected in the abundance of the proteins encoded by the partner genes, showing that it has far-reaching effects in yeast cellular networks beyond the immediate changes at the transcript level.

The functional importance of ‘anti-regulation’ in gene expression was demonstrated using a time course analysis of the cell cycle for three pairs involved in this process. In general, a rise in the expression of one gene occurred in parallel with falling levels of expression of the partner gene. This led to peaks in expression of one gene corresponding with troughs in the expression of the other gene, at various stages in the cycle. Moreover, we demonstrate that this cell-cycle expression pattern remains even when the convergent orientation of the pair is artificially converted into a tandem orientation. We have noted that the Proudfoot group conducted a systematic survey of the regulatory mechanism of convergent genes in *Schizosaccharomyces pombe* fission yeast. They demonstrated induced heterochromatinization for convergent genes occurring in a short period of G1-S phase, leading to down-regulation of the genes. This heterochromatinization event is mediated by the RNA inference (RNAi) pathway. In addition, most RNAi genes are themselves in convergent arrangement, resulting in auto-regulation between the convergent pairs [12], [25]. They modified arrangement of a pair of convergent genes by inserting an extra gene between the convergent pair, causing loss of the G1-S down regulation [25]. In contrast, here we have shown that modification of the convergent pair into a tandem arrangement does not result in loss of anti-regulation in budding yeast. This is likely due to fundamental differences in the biology of budding yeast (studied here) compared with fission yeasts [12], [25], as well as to differences in experimental procedures. First, as in metazoa, Dicer, Argonaut and RdRP, which are essential in the RNAi pathway, are conserved in *S. pombe* fission yeast, but lost in *S. cerevisiae* budding yeast [26]. Additionally, RNAi mediated regulation of convergent genes in fission yeast occurs through heterochromatinization, which requires several proteins including Clr4, RdRP, Tas3, Chp1 and Swi6, for which there are no known orthologs in *S. cerevisiae* budding yeast [26], [27]. These findings suggest there may be different mechanisms regulating the expression of convergent genes in *S. cerevisiae* budding yeast and *S. pombe* fission yeast. Second, we created a truly tandem arrangement of the gene pair by switching the orientation of one gene in the pair. Moreover, in the present study, the convergent genes had intact 3′-UTRs even after their orientation was converted into tandem. Third, the present study has focused on only those convergent genes with overlapping 3′-UTRs.

We have also demonstrated that the well-known environmental response of yeast cells to changes in nutrient availability is mediated through changes in expression of both the environment-responsive gene *ADE1*, and its convergent partner, *KIN3*. We conclude that the convergent organization of genes in the highly compact genome of *S. cerevisiae* is functionally significant and speculate that it represents a mode of gene expression regulation that may compensate for the absence of RNA-dependent regulatory systems.

The most widely accepted explanation for transcriptional interference between convergent gene pairs with opposite transcriptional direction is the transcriptional collision model [13]. The model proposes that during transcription, RNA polymerase progresses towards the 3′ end of each gene, and so the two processes on opposite strands will eventually clash; this will result in a negative correlation in transcript abundance between the convergent genes [13]. One prediction given by the model is that the transcriptional interference between convergent genes should be released when the genes are no longer in their native convergent arrangement. For the four pairs of convergent genes studied here, we have demonstrated for the first time that transcriptional interference between the partner genes can persist even when one of those genes is no longer expressed from its original location. Specifically, we have shown that ectopically over-expressing either the entire downstream gene, or its 3′-UTR alone, leads to suppressed expression of the convergent partner gene; this suppression is dependent on the 3′-UTR and is not observed when the ORF alone is ectopically expressed. This indicates that the 3′-UTR sequences are both necessary and sufficient to mediate transcriptional interference of convergent genes in the *S. cerevisiae* genome. Furthermore, transcriptional interference, at least for these four gene pairs, cannot be explained by the transcriptional collision model and must involve other, unexplored mechanisms.

## Materials and Methods

### Definition of ORFs with overlapping UTRs in the *S. cerevisiae* genome

The combination of high-resolution yeast transcriptome sequencing data [17] and genome sequence data enabled us to predict both 5′ and 3′ untranslated regions (UTRs) on a genome-wide basis. From all ORFs with either 5′ (4,835) or 3′ (5,212) UTRs, we identified UTRs overlapping by at least 1 base pair. From the mRNA sequencing dataset, we identified 645 ‘convergent’ ORF pairs with overlapping 3′-UTRs, 53 ‘divergent’ pairs with overlapping 5′-UTRs and 65 ‘consistent’ pairs with the same transcriptional direction. We also analyzed nascent transcript (NET-Seq) sequencing data [18] and strand specific RNA (ssRNA-Seq) sequencing data [19]. These three datasets were summarized in **Table S2**.

### Yeast strains and growth media

Haploid yeast strain YL1C was used as the wild type strain and is described in detail elsewhere [20]. Genetically modified strains shown in **Table 2** were constructed from the wild type following PCR-based protocols as follows. The upstream inhibited (group I) or downstream over-expressed (group II) strains were constructed by inserting an inhibitor or promoter segment accordingly into the upstream region of the target ORF. The terminator T_TEF_ and constitutive promoter P_ADH_ were amplified from the widely used plasmids pAG36 and pAG32 respectively. The downstream gene knock out (group III) or three ectopically expressed groups (V, VI and VII) were constructed through exchanging the relevant gene segments. The knocked out segments were extracted from the plasmid pAG36, and the downstream ORF and its 3′-UTR were amplified from the YL1C genomic DNA. For frame shift mutagenesis (group IV), one or two nucleotide bases were inserted into the coding sequence to generate premature termination in translation. In the terminator changed groups (VIII, IX and X), the 3′-overlapping regions of the convergent pairs were replaced by a T_ADH_-T_AOX1_ segment constructed through PCR-based fusion assembly [28]; the upstream gene inhibited (group IX) or downstream gene over-expressed (group X) strains were then constructed using the same methods used to construct group I and II strains. Finally, strains for the dual-luciferase assay (**Table S4**) were created as follows. Firstly, we used the reporter gene *Fluc* (firefly luciferase) vectored in the plasmid pGL3 (Promega) to replace the non-overlapping ORF regions of upstream genes of the convergent pairs, and then inserted *Rluc* (*Renilla* luciferase) vectored in the plasmid pRL-SV40 (Promega) into the genome as the internal control. *Fluc* and *Rluc* sequences can be found elsewhere [29], together with the promoter and terminator sequences used for constructing the internal control. All the genetic modifications constructed here were confirmed by Sanger sequencing. Primer sequences for molecular cloning are shown in Table S6.

Unless specified, both wild type and engineered strains were grown in the standard rich medium (YPD: 1% yeast extract, 2% polypepton, 2% glucose, plus 2% agar if necessary). In the adenine starvation environment, strains were grown in synthetic dropout medium (the adenine barren medium, SC-A: 0.67% yeast nitrogen base w/o amino acids, 2% glucose, 0.2% yeast synthetic dropout mixture without adenine, plus 2% agar if necessary). The synthetic complete medium (SC: 0.67% yeast nitrogen base w/o amino acids, 2% glucose, 0.2% yeast synthetic complete mixture, 2% agar if necessary) was used for the adenine rich environment control. Synthetic dextrose minimal medium (SD: 0.67% yeast nitrogen base w/o amino acids, 2% glucose) was used for the cell synchronization experiment.

### RNA extraction and real time quantitative PCR

Total RNA was extracted according to the hot acid phenol method as described in [30], followed by DNase I (Promega) cleanup to remove contaminating genomic DNA as described in [31]. The fractionated RNAs were used in 3′-RACE and real-time quantitative PCR. After reverse transcription, 1 µl cDNA templates were used for the quantitative PCR assay to compare expression levels of relevant genes [32]. For every tested strain, we took 3 independent clones as biological replicates for the PCR analysis. For each of the biological replicates, there were 3 technical replicates. Expression level was presented as the ratio of normalized target concentrations (ΔΔCt), as suggested elsewhere [33], [34].

### 3′-RACE sequencing

Protocols for 3′-RACE were implemented as described previously in [35]. In detail, the fractionated RNA was reverse transcribed using the Oligo (dT) anchor primer. The cDNA was then amplified with the 3′-end PCR anchor primer and gene-specific primers. After gel-purification, a nested PCR with the second anchor primer was conducted as necessary. We inferred the sequence reads to be the 3′-end of transcripts whenever poly-A appeared. The length of 3′-UTRs was counted up to and including the last nucleotide base before the poly-A. The sequence of the anchor primers is available from the commercial protocol (3′-RACE System for Rapid Amplification of cDNA Ends, Invitrogen).

### Nascent RNA expression analysis

Before newly transcribed RNA undergoes maturation processing (*i.e.* nascent RNA), the transcripts contain a segment which will be cleaved during the process of RNA maturation. Based on this structural feature of nascent RNA, together with the mRNA sequence and genomic sequence data, we designed nascent RNA specific reverse transcriptional primers to profile the expression of the nascent RNA. We tested for nascent expression of two pairs of convergent genes in the yeast genome using their nascent RNA specific reverse transcriptional primers as follows: ADE1-nascent-RT1 (5′-CACTGGCAAACAAGATATCG-3′), APT1-nascent-RT1 (5′-ATATTACTAT TGCATATGCAGGTC-3′), KIN3-nascent-RT1 (5′-AGAGACTGGCTTACTGCTAATAAG-3′), and UNG1-nascent-RT1 (5′-AAATGATATGTTTCACGTCCTG-3′).

### Dual luciferase assay

Luciferase assays followed the protocol described previously [36], [37] using the dual luciferase reporter (DLR) kits (Promega). In detail, the tested cells were grown in rich medium (YPD, 30°C) until the logarithmic phase (OD_600_ = 0.7–0.9). After washing and re-suspension in 1×PBS, the cultured cells were maintained in 100 µl 1× passive lysis buffer for 15 s. An aliquot of 5 µl was then extracted from the buffer for scoring luminescence measurements with 25 µl LAR II reagent from a Lumat LB 9507 (Berthold Technologies) set with 2 s delay time and 10 s measurement time. The same procedure was implemented after 25 µl Stop & Glo reagent was added. The protein expression level was recorded as the ratio of the firefly luciferase activity to the *Renilla* luciferase activity (*Fluc*/*Rluc*). For each test strain, at least three independent cultures were assayed.

### Cell-cycle synchronization

The protocol for cell-cycle synchronization was implemented as described previously [38]. The α pheromone-responsive strain YL1A (MATa, *bar1Δ*) was used to achieve cell-cycle synchronization in exactly the same genetic background as YL1C, which differs only in the mating type. The *BAR1* gene of YL1A was knocked out so that the strain would respond to a low density of α-factor [39], [40]. The logarithmic phase cells (OD_600_ = 0.5) were arrested and incubated for 1.5 hours once the α pheromone level reached 50 ng/ml. The cells were subsequently released from arrest by pelleting after repeated washing with pre-warmed ddH_2_O (30°C). The cells were then suspended in pre-warmed SD medium with Pronase E (0.1 mg/ml Pronase E, pH 6.4, 30°C) for 10 min. Subsequently, 15 ml of the prepared cell samples was taken every 20 minutes over the next two hours (approximating a complete cell-cycle) for extracting RNA, while the cell mass was kept at 30°C. In total, expression levels were measured at 6 time points.

### Nutrient-dependent growth test

Cells from the tested strains (D-K1 through to D-K7) were first cultured in rich medium (YPD) overnight, and then diluted to 3×10^5^ cells/ml (OD_600_ = 0.01). The diluted cultures were dropped on the adenine barren medium (synthetic dropout medium, SC-A) or the adenine rich medium (synthetic complete, SC) respectively. The cultures were further diluted in a gradient and 5 µl of every diluted culture was dropped on the test plates, which were incubated at 30°C for 48 hours.

### Profiling gene expression in single cells

5 ml yeast cells of wild-type YL1C were grown to the logarithmic phase in SD medium. Cells were then washed twice with sterilized water, and re-suspended in 5 ml ultrapure water. Cells were counted using a hemocytometer and diluted with reverse transcription buffer (Invitrogen) to only one cell per 7 µl. 7 µl aliquots were deposited into each well of a 384-well cell plate (Corning). We identified wells containing only a single cell (single-cell well) using an inverse microscope. The method used to profile gene expression in a single cell was slightly modified from the documented protocol [41], briefly described as follows. 1 µl mixture of lyticase (2 µg/µl, Sigma), DNase I (1 unit/µl, Promega) and RNase OUT (2 uint/µl, Invitrogen) were added to the single-cell well, and these wells were incubated for 15 min at 30°C, then for 15 min at 37°C to lyse the cells. Another 1 µl mixture of Proteinase K (0.1 µg/µl) and human total RNA spike-in control (10 ng/µl) was added to the single-cell lysate and incubated for 10 min at 65°C. Each microliter of the reverse transcriptase mixture (1 mM dNTP, 5 µM oligo-dT, 5 µg/µl BSA, and 20 unit/µl SuperScript III) was used to initiate the aforementioned single-cell reverse transcription. To enhance the template level for quantitative PCR assay, we first performed a 15-cycle nested PCR for *KIN3* and *ADE1* genes, with human *ACT1* as the spike-in control. The nested PCR primers in 5′ to 3′ direction were GCCACAACATACGTCGGTACA and AGGATTTTTTCAATGTTTGTCAGC for *KIN3*, TCTTCACCCCATCGACCAA and CAGTAAGCCAGTCTCTTAAAAATTGC for *ADE1*, GCACAGAGCCTCGCCTTT and CGTGCTCGATGGGGTACTTC for *ACT1*. A 0.5 µl aliquot of PCR product was used as the template for the next round of RT-PCR, using the following primers: GCCACAACATACGTCGGTACA and GGGAGTATGGTTGGTCCATCA for *KIN3*, TCTTCACCCCATCGACCAA and GGGCAGGAGAGATGTTTTCG for *ADE1*, GCACAGAGCCTCGCCTTT and GTTGTCGACGACGAGCG for *ACT1*.

### Microarray datasets and analysis

Microarray expression datasets were downloaded from the NCBI Gene Expression Omnibus (GEO) database under the series accession numbers GSE19213, GSE13684 and GSE5185 (http://www.ncbi.nlm.nih.gov/geo). These datasets were collected from an *S. cerevisiae* wild type strain BY4741 cultivated in seven different environments (**Table S3**). The tested cells were harvested at the early log (or exponential) phase (OD_600_ = 0.3–0.4). Total RNA was extracted and processed according to the manufacturer's instructions (www.affymetrix.com). The Yeast Genome 2.0 (YEAST 2.0) microarray, which contained 5,744 probe sets interrogating all annotated ORFs in the *S. cerevisiae* genome, was employed to profile transcript abundance of the ORFs. Transcript abundance was extracted from raw hybridization signal intensities of each probe set using the Robust Multichip Average (RMA) method implemented in R [42]. The expression value for each ORF was log_2_-transformed prior to analysis.

## Supporting Information

Figure S1 Status of the 645 convergent gene pairs with overlapping 3′-UTRs as predicted from mRNA-Seq data, in both nascent RNA sequencing data (A) and strand-specific RNA sequencing data (B).
(TIF)

Figure S2 Correlation in expression between convergent gene pairs with overlapping 3′- UTRs across seven growth environments. Box plots showing the expression levels of 645 convergent gene pairs with overlapping 3′-UTRs across seven growth conditions described in **Table S3**. Above each box is the Pearson correlation coefficient (*r*) between partner genes and the corresponding *P* value.
(TIF)

Figure S3 Mapped reads of convergent gene pairs *APT1/UNG1*, *ADE1/KIN3*, *SHM1/YPT10*, and *AXL2/REV7* from nascent RNA sequencing data (a–d) or from strand-specific RNA sequencing datasets (e–h).
(TIF)

Figure S4 Correlated expression of the convergent gene pair, *KIN3* and *ADE1*, measured in single cells.
(TIF)

Table S1 ORFs with overlapping 3′-UTRs identified in the budding yeast *S. cerevisiae* genome.
(DOC)

Table S2 Summary information of RNA-Seq, NET-Seq and ssRNA-Seq datasets.
(DOC)

Table S3 Seven environmental treatments used in yeast microarray analysis studies.
(DOC)

Table S4 Genetically modified strains used in the dual luciferase assays.
(DOC)

Table S5 Sequence of the convergent gene pair, *KIN3* and *ADE1*, in wild type and two genetically modified strains.
(DOC)

Table S6 Primers using in RT-PCR, 3′-RACE, and molecular cloning.
(DOC)
