# Supplementary figures and images for: 3′ Untranslated Regions Mediate Transcriptional Interference between Convergent Genes Both Locally and Ectopically in Saccharomyces cerevisiae
Source: PLoS Genet. 2014 Jan 23;10(1):e1004021. doi: 10.1371/journal.pgen.1004021 (PMC3900390; doi:10.1371/journal.pgen.1004021)

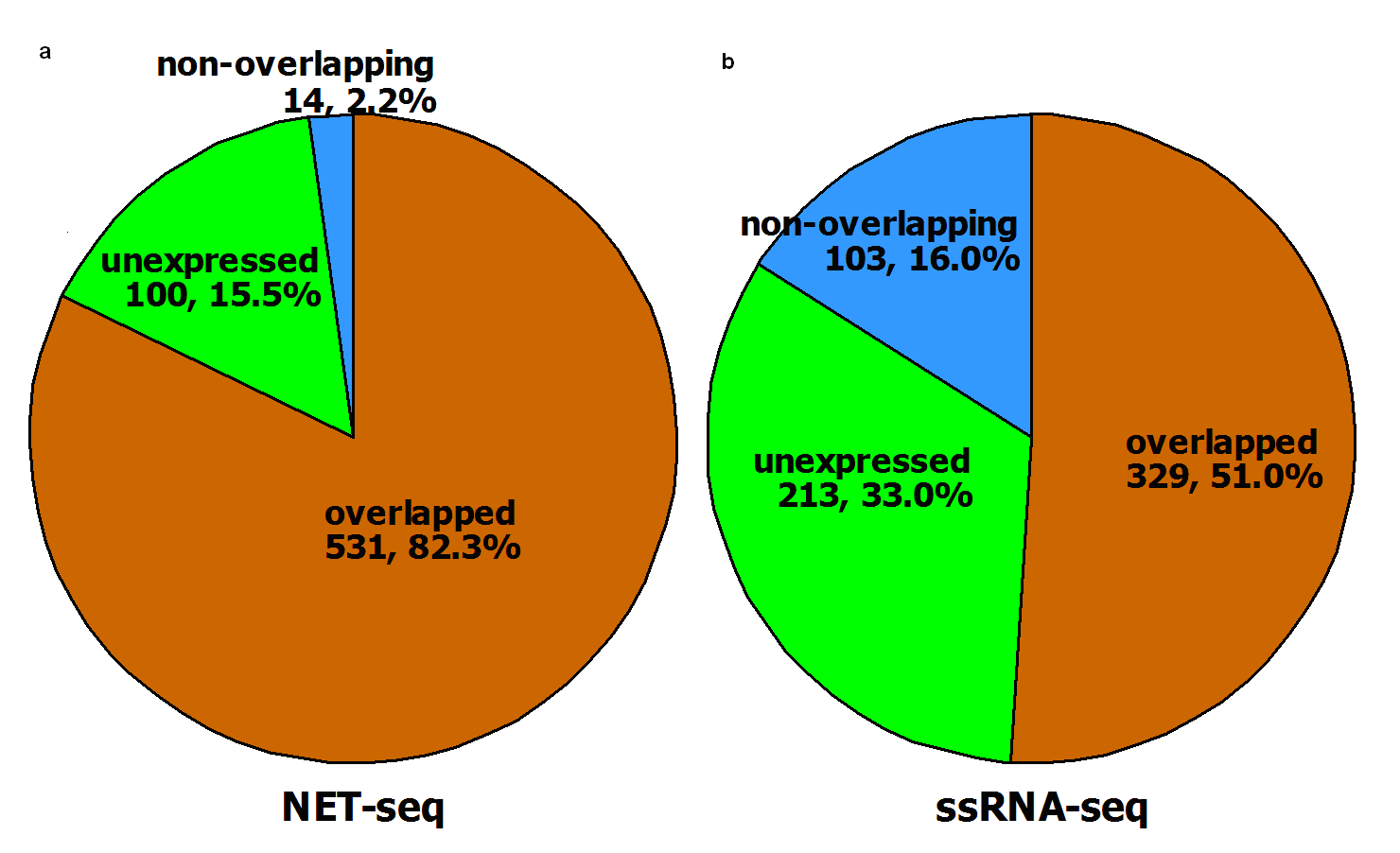

Supplement: Figure S1 — Status of the 645 convergent gene pairs with overlapping 3′-UTRs as predicted from mRNA-Seq data, in both nascent RNA sequencing data (A) and strand-specific RNA sequencing data (B). (TIF) [file pgen.1004021.s001.tif]

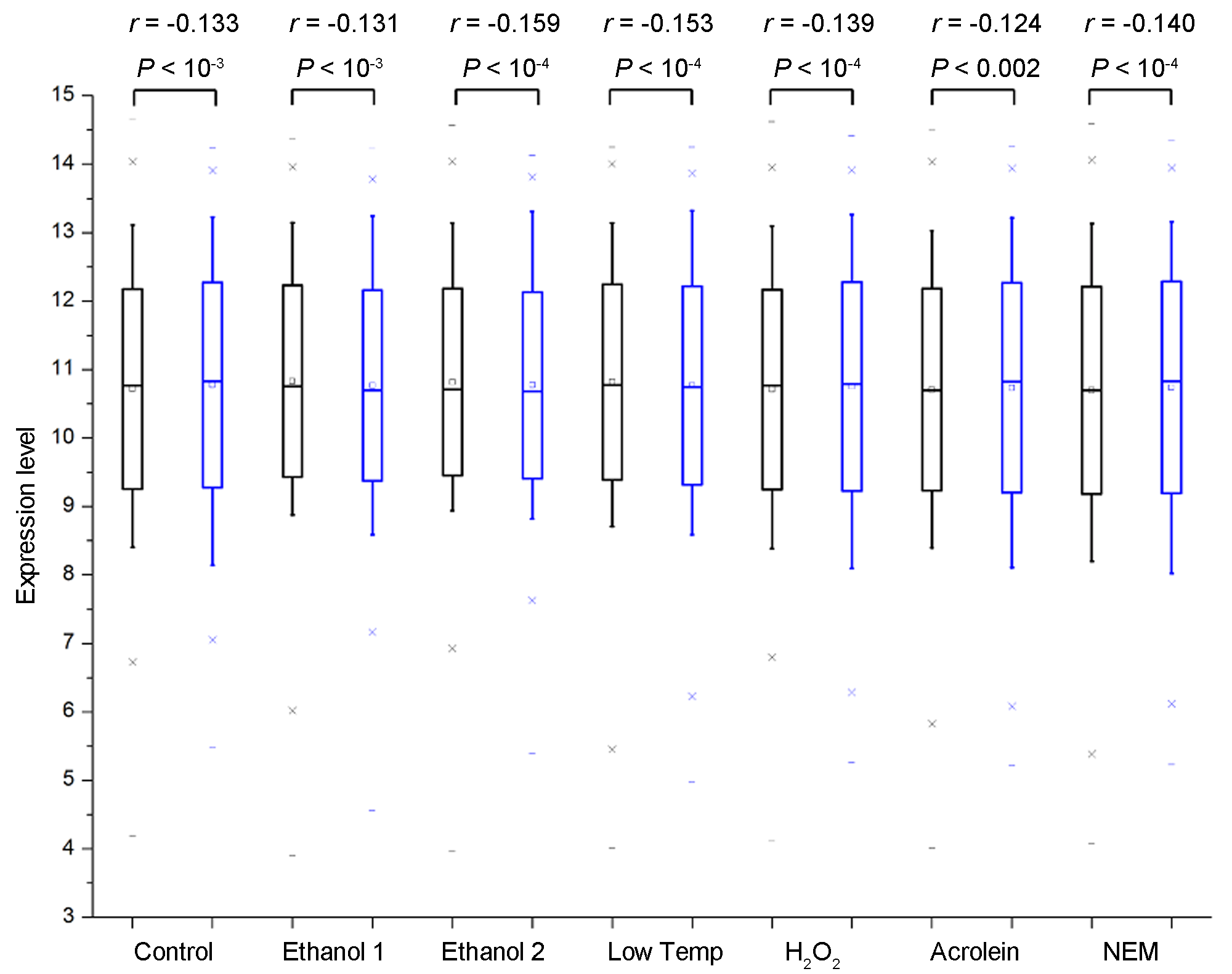

Supplement: Figure S2 — Correlation in expression between convergent gene pairs with overlapping 3′- UTRs across seven growth environments. Box plots showing the expression levels of 645 convergent gene pairs with overlapping 3′-UTRs across seven growth conditions described in Table S3. Above each box is the Pearson correlation coefficient (r) between partner genes and the corresponding P value. (TIF) [file pgen.1004021.s002.tif]

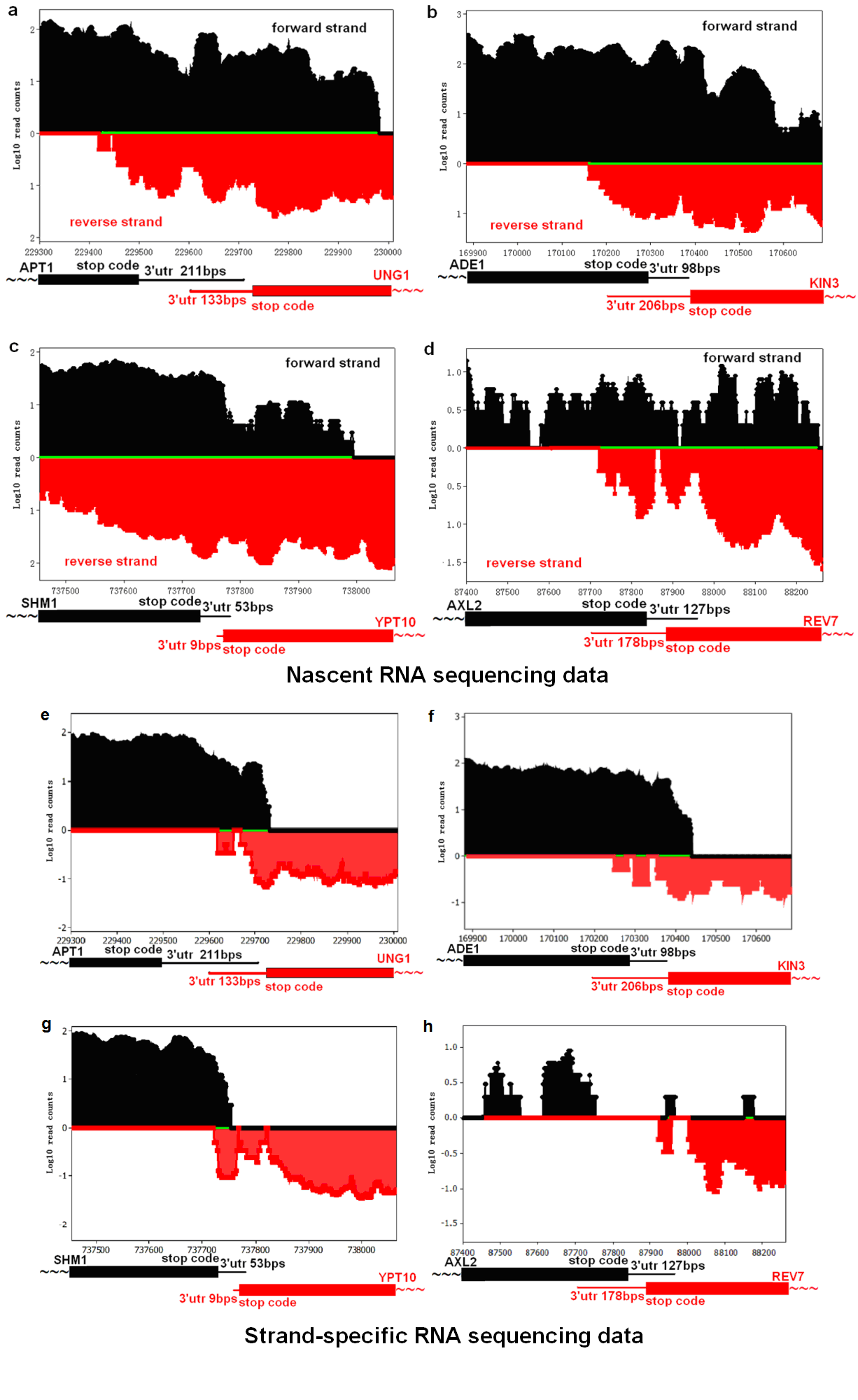

Supplement: Figure S3 — Mapped reads of convergent gene pairs APT1/UNG1, ADE1/KIN3, SHM1/YPT10, and AXL2/REV7 from nascent RNA sequencing data (a–d) or from strand-specific RNA sequencing datasets (e–h). (TIF) [file pgen.1004021.s003.tif]

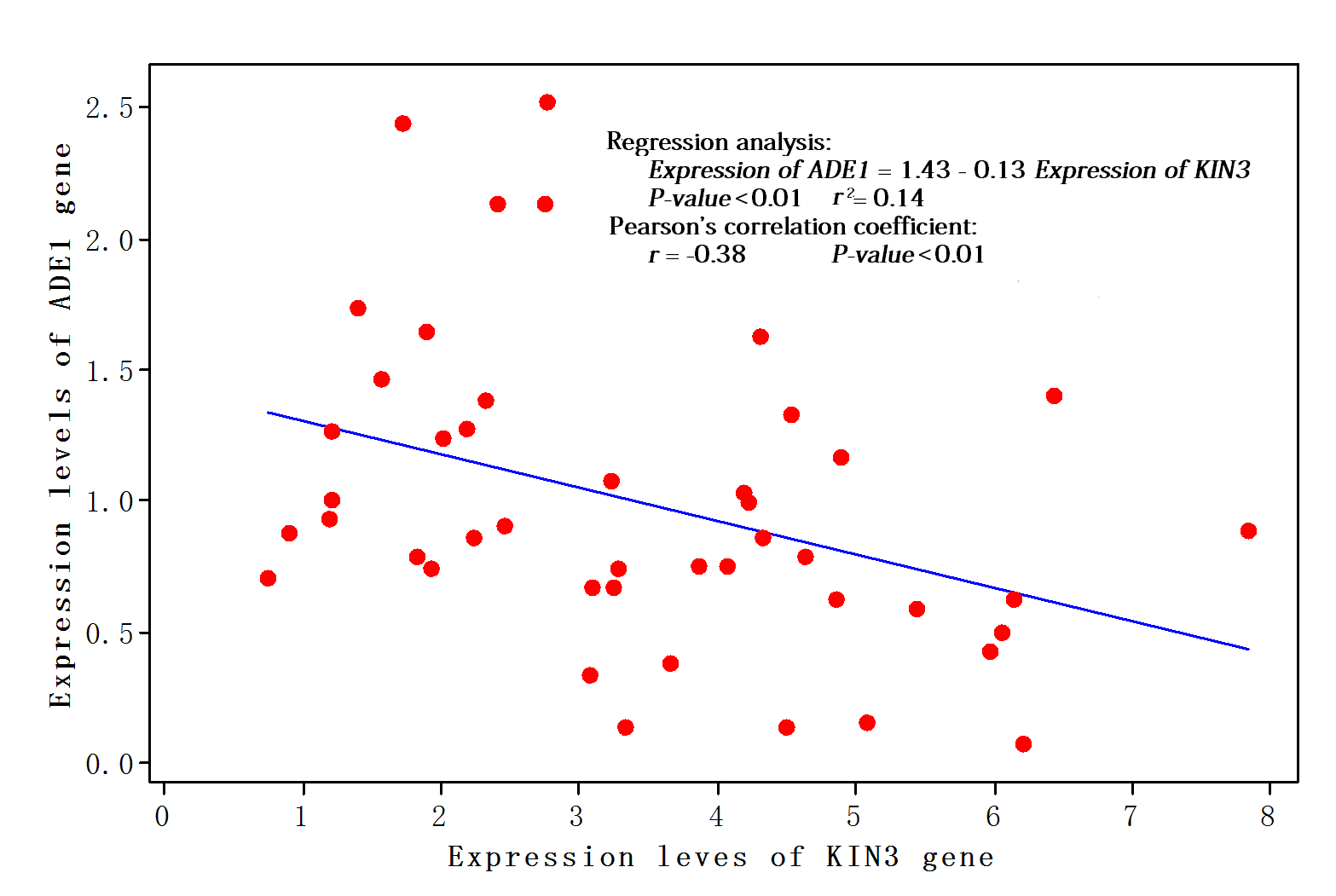

Supplement: Figure S4 — Correlated expression of the convergent gene pair, KIN3 and ADE1, measured in single cells. (TIF) [file pgen.1004021.s004.tif]
