# Supplementary material for: 3′ Untranslated Regions Mediate Transcriptional Interference between Convergent Genes Both Locally and Ectopically in Saccharomyces cerevisiae
Source: PLoS Genet. 2014 Jan 23;10(1):e1004021. doi: 10.1371/journal.pgen.1004021 (PMC3900390; doi:10.1371/journal.pgen.1004021)
Supplement: Table S1 — ORFs with overlapping 3′-UTRs identified in the budding yeast S. cerevisiae genome. (DOC) [file pgen.1004021.s005.doc]

Table S1. ORFs with overlapping 3'-UTRs identified in the budding yeast *S. cerevisiae* genome.

| Number (%) of gene pairs with overlapping 3'-UTR | | |
| --- | --- | --- |
| Convergent | Consistent | Divergent |
| 654 (20%*) | 65 (2%) | 53 (1.6%) |

*Percentages are given as the percentage of the total (n = 5,770) genes in the *S. cerevisiae* genome. We have defined convergent gene pairs as those that overlap at their 3’ ends, divergent gene pairs as those that overlap at their 5’ ends and consistent gene pairs as those that overlap and are transcribed in the same direction. Overlap is defined by at least 1bp.
