# Supplementary material for: 3′ Untranslated Regions Mediate Transcriptional Interference between Convergent Genes Both Locally and Ectopically in Saccharomyces cerevisiae
Source: PLoS Genet. 2014 Jan 23;10(1):e1004021. doi: 10.1371/journal.pgen.1004021 (PMC3900390; doi:10.1371/journal.pgen.1004021)
Supplement: Table S2 — Summary information of RNA-Seq, NET-Seq and ssRNA-Seq datasets. (DOC) [file pgen.1004021.s006.doc]

Table S2: Summary information of RNA-Seq, NET-Seq and ssRNA-Seq datasets.

|  | RNA-seq | NET-seq | ssRNA-seq |
| --- | --- | --- | --- |
| Species | *S. cerevisiae* | *S. cerevisiae* | *S. cerevisiae* |
| Strain sequenced | BY4741 | BY4741 | BY4741 |
| Culture condition | YPAD at 30°c | YEPD at 30°c | YEPD at 30°c |
| RNA isolation | mature mRNA with  poly A+ | nascent RNA with RNAPII elongation complex | mature mRNA with  poly A+ |
| Sequencing platform | Illumina 1G high throughput sequencing | Illumina GA2 | Illumina GA |
| Sequencing strategy | Double-stranded cDNA | Strand-specific | Strand-specific |
| Read type | Single-end | Single-end | Single-end |
| Read length | 33 bps | 40 bps | 36 bps |
| Total number of reads | 29,912,517 | 63,828,233 | 12,236,284 |
| Reference | [17] | [18] | [19] |
