# Supplementary material for: 3′ Untranslated Regions Mediate Transcriptional Interference between Convergent Genes Both Locally and Ectopically in Saccharomyces cerevisiae
Source: PLoS Genet. 2014 Jan 23;10(1):e1004021. doi: 10.1371/journal.pgen.1004021 (PMC3900390; doi:10.1371/journal.pgen.1004021)
Supplement: Table S3 — Seven environmental treatments used in yeast microarray analysis studies. (DOC) [file pgen.1004021.s007.doc]

Table S3. Seven environmental treaments used in yeast microarray analysis studies.

| ID | Treatment name and description | Reference and source |
| --- | --- | --- |
| 1 | Control: YPD medium at 30^o^C | **Ouyang *et al.,* (2011); GSE19213** |
| 2 | Ethanol 1: 0% EtOH, 20 g/L glucose at 30^o^C | Alper *et al.*, (2006); GSE5185 |
| 3 | Ethanol 2: 5% EtOH, 60 g/L glucose at 30^o^C | Alper *et al.*, (2006); GSE5185 |
| 4 | Low Temperature: YPD medium at 25^o^C | Zhu *et al.*, (2009); GSE13684 |
| 5 | H_2_O_2_: 0.3mM H_2_O_2_, YPD medium at 30^o^C | **Ouyang *et al.,* (2011); GSE19213** |
| 6 | Acrolein: 0.05mM acrolein, YPD medium at 30^o^C | **Ouyang *et al.,* (2011); GSE19213** |
| 7 | NEM^*^: 0.016 mM NEM, YPD medium at 30^o^C | **Ouyang *et al.,* (2011); GSE19213** |

**All studies used the yeast strain BY4741. Datasets are available from the Gene Expression Omnibus (GEO) database with the given GSE accession numbers. *NEM denotes N-ethylmaleimide.**
