# Supplementary material for: 3′ Untranslated Regions Mediate Transcriptional Interference between Convergent Genes Both Locally and Ectopically in Saccharomyces cerevisiae
Source: PLoS Genet. 2014 Jan 23;10(1):e1004021. doi: 10.1371/journal.pgen.1004021 (PMC3900390; doi:10.1371/journal.pgen.1004021)
Supplement: Table S4 — Genetically modified strains used in the dual luciferase assays. (DOC) [file pgen.1004021.s008.doc]

Table S4. Genetically modified strains used in the dual luciferase assays.

| Modified strain | Modification targeted | Modifications made | Internal control |
| --- | --- | --- | --- |
| fpuYL1C  fP-U2  fP-U6  fP-U7 | DLR wild type control*  Downstream gene over-expressed  Downstream gene with ectopic ORF  Downstream gene with ectopic 3’-UTR | *apt1^ORF^*::*Fluc^ORF^* (Knocking out *APT1^ORF^* with Fluc ORF) | *ura3^ORF^*::*Rluc* (Knocking out *URA3^ORF^* with Rluc^ORF^ constitutively expressed by *SPT15*-promoter and terminated by *ADH*-terminator) |
| fdkYL1C  fD-K2  fD-K6  fD-K7 | DLR wild type control  Downstream gene over-expressed  Downstream gene with ectopic ORF  Downstream gene with ectopic 3’-UTR | *ade1^ORF^*::*Fluc^ORF^* (Knocking out *ADE1^ORF^* with Fluc ORF) | Same as above. |

*DLR denotes the Dual Luciferase Reporter assay. All strains have DLR background.
