# Supplementary material for: 3′ Untranslated Regions Mediate Transcriptional Interference between Convergent Genes Both Locally and Ectopically in Saccharomyces cerevisiae
Source: PLoS Genet. 2014 Jan 23;10(1):e1004021. doi: 10.1371/journal.pgen.1004021 (PMC3900390; doi:10.1371/journal.pgen.1004021)
Supplement: Table S5 — Sequence of the convergent gene pair, KIN3 and ADE1, in wild type and two genetically modified strains. (DOC) [file pgen.1004021.s009.doc]

**Table S5 Sequence of the convergent gene pair, KIN3 and ADE1, in wild type and two genetically modified strains tested in Figure 5.**

**WT strain**

CAGTAATTCAAAAGTGGAAGGCCTGGTTCTTAAGTTCACATCTATCATTGAATGTATTATGGCATTAAGCCCTCTAGAGTAATACTCAGGGACGGTGTCACATTTCCCGTTTTTAATCTTAGTTTGTAGCTCGAGATAATTTTTTGCCTGAAATGGGGGGTGCAACGAACACATCTCAAAAATAACACAACCTAGTGACCAGATGTCGGATAGTGGGGAGTATGGTTGGTCCATCAACACTTCAGGCGACATGTAATATGGTGTACCGACGTATGTTGTGGCAAATTGAATACTAGTTTCCAGAGATTTGGCTAACCCAAAATCACCTAACTTTACCACAACTTGACTATAGTCCATAGGGCTCCCCCTTTTCCCTGAATTCACTCTATGGTCTCTGTAATAATTACTATTCACTTCCTCGTGACCGTCTACTTGTTCATTAATATTGTAATCGCTATCATCATAGCTTAAGAATATATTTCCTGGTTTCAGATCACGATGGATAACGATGTTTTTGCCTTTTACCGGTGGTTTCATCCGGTCATATATTGTGGTCAAAGTTGGCAATTCAACACCATAATGACATTTATAGAGCGCAGTCAATAATTGGGCCAGGATACCCCACACAATTTTTTCTGGTATATATTTATGCTCCTGTTTGTAGTGCTTAATCATCTGGGATAAATCACCCCTGGAACAGTATTCCATATAAAGGTATAACACTTCTTTTTGTTCATCGAAATCCCAGTTATAAAATTCTACAATATTTTCATGCTTCAACTGCGATAGAATGCTACATTCAGCGATCAGCTGTTGTCTCTCTTTGCTATTCATATGGCCATATTTGATATCCTTTCTAACCAAAAGTTTCTTGGTAGGTATATGGATGACTTTTCGTACAGACCCAAATGAACCTCTCCCAATTTCTTCGAGAACTTGGTATTCTGACCCTGGTGGGTGTCCCTGCTGCTGCTGAGGACTACGGTATTCTTGGAAAAACTGTCGTCTATGCATACTCACACAGAGAATTGATTCAATTATCAAATAGCACTCTCATTGAAATTAGTATTGTGAATCTTGCTCTTTTCATGTTATATGATTTGATATTCTTTTGAAAAGTCGCTTTTATTTACGTTTAACCTAATTAGGAAACGTAATGAAAAAAATTCAGAAACCTTAAAAAAAAAAACTTGGCTGTAACCTATCGGAAGACTGTGCCACTGCAATCATGTCAGATATCGTATTTCAGATTTATTGATCTATAGCTAGAAACATT-AACAAAATGCGCTTTGAGTCGTTCATACATTTAATCCCCAATTGAAAAAAAAAAGAAAAGAAAAAAAGCATATATATGTATATGCTTTTTTATCATTACTGGCCTC

**Tandem URA3**

CAGTAATTCAAAAGTGGAAGGCCTGGTTCTTAAGTTCACATCTATCATTGAATGTATTATGGCATTAAGTAATGTGGCTGTGGTTTCAGGGTCCATAAAGCTTTTCAATTCATCTTTTTTTTTTTTGTTCTTTTTTTTGATTCCGGTTTCTTTGAAATTTTTTTGATTCGGTAATCTCCGAGCAGAAGGAAGAACGAAGGAAGGAGCACAGACTTAGATTGGTATATATACGCATATGTGGTGTTGAAGAAACATGAAATTGCCCAGTATTCTTAACCCAACTGCACAGAACAAAAACCTGCAGGAAACGAAGATAAATCATGTCGAAAGCTACATATAAGGAACGTGCTGCTACTCATCCTAGTCCTGTTGCTGCCAAGCTATTTAATATCATGCACGAAAAGCAAACAAACTTGTGTGCTTCATTGGATGTTCGTACCACCAAGGAATTACTGGAGTTAGTTGAAGCATTAGGTCCCAAAATTTGTTTACTAAAAACACATGTGGATATCTTGACTGATTTTTCCATGGAGGGCACAGTTAAGCCGCTAAAGGCATTATCCGCCAAGTACAATTTTTTACTCTTCGAAGACAGAAAATTTGCTGACATTGGTAATACAGTCAAATTGCAGTACTCTGCGGGTGTATACAGAATAGCAGAATGGGCAGACATTACGAATGCACACGGTGTGGTGGGCCCAGGTATTGTTAGCGGTTTGAAGCAGGCGGCGGAAGAAGTAACAAAGGAACCTAGAGGCCTTTTGATGTTAGCAGAATTGTCATGCAAGGGCTCCCTAGCTACTGGAGAATATACTAAGGGTACTGTTGACATtGCGAAGAGCGACAAAGATTTTGTTATcGGCTTTATTGCTCAAAGAGACATGGGTGGAAGAGATGAAGGTTACGATTGGTTGATTATGACACCCGGTGTGGGTTTAGATGACAAGGGAGACGCATTGGGTCAACAGTATAGAACCGTGGATGATGTGGTCTcTACAGGATcTGACATTATTATTGTTGGAAGAGGACTATTTGCAAAGGGAAGGGATGCTGAGGTAGAGGGTGAACGTTACAGAAAAGCAGGCTGGGAAGCATATTTGAGAGGATGCGGCCAGCAAAACTAAAAAACTGTATTATAAGTAAATGCATGTATACTAAACTCACAAATTAGAGCTTCAATTTAATTATATCAGTTATTACCCGGGAATcTCGGTCGTAATGATTTCTATAATGACGAAAAAAAAAAATTGGAAAGAAAAAGCTTCATGGCCTTTATAAAAAGGAACTATCCAATACCTCGCCAGAACCAAGTTAATCCCCAATtGAAAAAAAAAGAAAAGAAAAAAAGCATATATATGTATATGCTTTTTTATCATTACTGGCCTC

**Tandem KIN3**

ATATATGAGGTAGACGCTGGTACGTTGCTGTTTGTCGCTACGGATCGTATCTCTGCATATGACGTTATTATGGAAAACAGCATTCCTGAAAAGGGGATCCTATTGACCAAACTGTCAGAGTTCTGGTTCAAGTTCCTGTCCAACGATGTTCGTAATCATTTGGTCGACATCGCCCCAGGTAAGACTATTTTCGATTACCTACCTGCAAAATTGAGCGAACCAAAGTACAAAACGCAACTAGAAGACCGTTCTCTATTGGTTCACAAACATAAACTAATTCCATTGGAAGTAATTGTCAGAGGCTACATCACCGGATCTGCTTGGAAAGAGTACGTAAAAACAGGTACTGTGCATGGTTTGAAACAACCTCAAGGACTTAAAGAATCTCAGGAGTTCCCAGAACCAATCTTCACCCCATCGACCAAGGCTGAACAAGGTGAACATGATGAAAACATCTCTCCTGCCCAGGCCGCTGAGCTGGTGGGTGAAGATTTGTCACGTAGAGTGGCAGAACTGGCTGTAAAACTGTACTCCAAGTGCAAAGATTATGCCAAGGAGAAGGGCATCATCATCGCAGACACCAAATTCGAATTCGGTATTGACGAAAAGACCAATGAAATTATTCTAGTGGACGAGGTGCTAACGCCAGACTCCTCTAGATTCTGGAACGGTGCCTCTTATAAGGTAGGAGAATCCCAAGATTCTTACGATAAGCAATTTTTAAGAGACTGGCTTACTGCTAATAAGTTGAACGGTGTTAACGGCGTCAAAATGCCCCAAGACATTGTCGACAGGACAAGGGCCAAATATATAGAGGCTTATGAAACATTGACAGGGTCTAAATGGTCTCACTAACGTGATTTACATATACTACAAGTCGCCAGTGTAACTCCTCACTGAATATGATTCATACATACCCGTATGTATTAATGTATAAATGTTCTCAGAGCAAATTTTATCGATATCTTGTTTGCCAGTGGTATGCAGGTTTGGCAAATTTTTTACCATAATATCCGTTTATAGATTCTGGAACCTTACCAACTTTCTTACCGCTAATTACTTCCCTGGCTCGCTCCTCCACTGCCTGGGTAAATTGTTCCTTCAACTGACTCAGTTCTCTTTCGTATTCAATAGCTTGCTTCTCGAGGATTTTTTCAATGTTTGTCAGCTCATTTTCATAGTCCAGTAACTTCCTTTCAAATCTCTCTAATTGCAACGACTTTCTTGCAGTTCGTATCTGAATATCTTGCAGTAATTCAAAAGTGGAAGGCCTGGTTCTTAAGTTCACATCTATCATTGAATGTTTATGGCATTAAGATGACGTCGGGATTAAATGTATGAACGACTCAAAGCGCATTTTGTTAATGTTTCTAGCTATAGATCAATAAATCTGAAATACGATATCTGACATGATTGCAGTGGCACAGTCTTCCGATAGGTTACAGCCAAGTTTTTTTTTTTAAGGTTTCTGAATTTTTTTCATTACGTTTCCTAATTAGGTTAAACGTAAATAAAAGCGACTTTTCAAAAGAATATCAAATCATATAACATGAAAAGAGCAAGATTCACAATACTAATTTCAATGAGAGTGCTATTTGATAATTGAATCAATTCTCTGTGTGAGTATGCATAGACGACAGTTTTTCCAAGAATACCGTAGTCCTCAGCAGCAGCAGGGACACcCACCAGGGTCAGAATACCAAGTTCTCGAaGAAATTGGGAGAGGTTCATTTGGGTCTGTACGAAAAGTCATCCATATACCTACCAAGAAACTTTTGGTTAGAAAGGATATCAAATATGGCCATATGAATAGCAAAGAGAGACAACAGCTGATCGCTGAATGTAGCAtTCTATCGCAGTTGAAGCATGAAAATaTTGTAGAATTTTATAACTGGGATTTCGATGAACAAAAAGAAGTGTTATACCTTTATATGGAATACTGTTCCAGGGGTGATTTATCCCAGATGATTAAGCACTACAAACAGGAGCATAAATATATACCAGAAAAAATTGTGTGGGGTATCCTGGCCCAATTATTGACTGCGCTCTATAAATGTCATTATGGTGTTGAATTGCCAACTTTGACCACAATATATGACCGGATGAAACCACCGGTAAAAGGCAAAAACATCGTTATCCATCGTGATCTGAAACCAGGAAATATATTCTTAAGTTATGATGATAGCGATTACAATATTAATGAACAAGTAGACGGTCACGAGGAAGTGAATAGTAATTATTACAGAGACCATAGAGTGAATTCAGGGAAAAGGGGGAGCCCTATGGACTATAGTCAAGTTGTGGTAAAGTTAGGTGATTTTGGGTTAGCCAAATCTCTGGAAACTAGTATTCAATTTGCCACAACATACGTCGGTACACCATATTACATGTCGCCTGAAGTGTTGATGGACCAACCATACTCCCCACTATCCGACATcTGGTCACTAGGTTGTGTTATTTTTGAGATGTGTTCGTTGCACCCCCCATTTCAGGCAAAAAATTATCTCGAGCTACAAACTAAGATTAAAAACGGGAAATGTGACACCGTCCCTGAGTATTACTCTAGAGGGCTTAATGCCATAATACATTCAATGATAGATGTGAACTTAAGAACCAGGCCTTCCACTTTTGAATTACTGCAAGATATTCAGATACGAACTGCAAGAAAGTCGTTGCAATTAGAGAGATTTGAAAGGAAGTTACTGGACTATGAAAATGAGCTGACAAACATTGAAAAAATCCTCGAGAAGCAAGCTATTGAATATGAAAGAGAACTGAGTCAGTTGAAGGAACAATTTACCCAGGCAGTGGAGGAGCGAGCCAGGGAAGTAATTAGCGGTAAGAAAGTTGGTAAGGTTCCAGAATCTATAAACGGATATTATGGTAAAAAATTTGCCAAACCTGCATACCACTGGCAAACAAGATATCGATAAAATTTGCTCTGAGAACATTTATACATTAATACATACGGGTATGTATGAATCATATTCAGTGAGGAGTTACACTGGCGACTTGTAGTATATGTAAATCACGTTAGTGAGACCATTTAGACCCTGTCAATGTTTCATAAGCCTCTATATATTTGGCCCTTGTCCTGTCGACaATGTCTTGGGGCATTTTGACGCCGTTAACACCGTTCAACTTATTAGCAGTAAGCCAGTCTCTTAACAATTGAAAAAAAAAAGAAAAGAAAAAAAGCATATATATGTATATGCTTTTTTATCATTACTGGCCTCTTTAAATTCAAAAaCTTTTCTGATCTCTTTTCCAAACAGATGCGTTTTCAGtATTGGAAGGTTCACAaTTCTATATATAGTGTTAATGTAATGCTGTATTATTTCTCtATAtATGTAtGTATGCACATGCAATTCCTACATTATGTTTGAAATGTtGTAATGGGgAcGgAAaAGccGTCACTTTtATCTTtGgAgGATCGCAaaTTACTACGCTCATCTTTTGTTGGAGA
