# Supplementary material for: 3′ Untranslated Regions Mediate Transcriptional Interference between Convergent Genes Both Locally and Ectopically in Saccharomyces cerevisiae
Source: PLoS Genet. 2014 Jan 23;10(1):e1004021. doi: 10.1371/journal.pgen.1004021 (PMC3900390; doi:10.1371/journal.pgen.1004021)
Supplement: Table S6 — Primers using in RT-PCR, 3′-RACE, and molecular cloning. (DOC) [file pgen.1004021.s010.doc]

Table S6. Primers used in RT-PCR, 3’-RACE, and molecular cloning in the paper.

* The lowercase in the primers means sites that can be cut by restriction enzymes.

| **Primers in RT-PCR assay** | |
| --- | --- |
| RTshm-f | TGCGGCGCATATTTGATG |
| RTshm-r | AACATTGGCTGCCACCAAAC |
| RTypt-f | GCTCAGGGAACGCAGGTAATT |
| RTaxl-f | CGTCAAGGGATGTCACTATGTCTT |
| RTaxl-r | ACGGGAGAAGGGCTAATATTGC |
| RTrev-f | CGATGACACAATAACATTTGAAGCA |
| RTrev-r | CCTGTTTCTGTCCAACTTATGTCCTA |
| RTrev-f2 | GGGCCTTTGATTATTCATCAGTTT |
| RTrev-r2 | TCTTCATATTGAGAATACACTCCATTCA |
| RTapt-f | ATTATATCGTCGGGTTGGAA |
| RTapt-r | GGGCCAAAGCTAAAGTTGGT |
| RTung-f | AGGCGCCCAATAAGAAAGGT |
| RTung-r | GCGCCATTTGTTATGGTCATG |
| RTung-f2 | AATACCCGCGGAGAGAAAATG |
| RTung-r2 | TGCCTCCTGAACTTCTCTTAATGA |
| RTade-f2 | TCTTCACCCCATCGACCAA |
| RTade-r2 | GGGCAGGAGAGATGTTTTCG |
| RTkin-f | GCCACAACATACGTCGGTACA |
| RTkin-r | GGGAGTATGGTTGGTCCATCA |
| RTkin-f2 | TTTACCCAGGCAGTGGAGGA |
| RTkin-r2 | ACCTTACCAACTTTCTTACCGCTA |
| RTura3-f | GCGGCGGAAGAAGTAACAAA |
| RTura3-r | AGCCCTTGCATGACAATTCTG |
| SYACT1F | TCGTTCCAATTTACGCTGGTT |
| SYACT1R | CGGCCAAATCGATTCTCAA |
| **3’-RACE sequencing assay** | |
| Oligo(dT) anchor | GCGAGCACAGAATTAATACGACTCACTATAGGT12VN |
| PCR anchor | GCGAGCACAGAATTAATACGACT |
| Second anchor | CGCGGATCCGAATTAATACGACTCACTATAGG |
| SHM1 (out) | TGCGGCGCATATTTGATG |
| SHM1 (in) | CAGCCAATGTTGTCCCATCTCCA |
| YPT10 (out) | GCTCAGGGAACGCAGGTAATT |
| YPT10 (in) | GGGTCTGCCGTATGTGGCCGTAA |
| AXL2 (out) | CTCAAACCACAGTGCGAAT |
| AXL2 (in) | CGTCAAGGGATGTCACTATGTCTT |
| REV7 (out) | CTAAATTAACGCACGTTTACAG |
| REV7 (in) | CGATGACACAATAACATTTGAAGCA |
| APT1 (out) | ATTATATCGTCGGGTTGGAA |
| APT1 (in) | TGGCCCTAGGTGTTGGTTTC |
| UNG1 (out) | CGTTGGAAGCGGTAGTAAG |
| UNG1 (in) | TAACTCACTGGGCTTCACAA |
| ADE1 (out) | GCTAAGGAGAAGGGCATCA |
| ADE1 (in) | ACGGTGTTAACGGCGTCAA |
| KIN3 (out) | GCCACAACATACGTCGGTACA |
| KIN3 (in) | TGTTGATGGACCAACCATACTCCC |
| **For creating genetically modified strains (Groups I-X)** | |
| Inserting an inhibitor (group I) | |
| PSshm-f | AGCCGACATTTCCACATCGAGTCTCATAGTCTTTCAATCAGCTGAAGCTTCGTACGC |
| PSshm-r | TGCCATACATTTGGCCAATGCAGAAGCTCTGGGAAACATGCATAGGCCACTAGTGGATC |
| PSapt-f | CCTGCTTCAAAGAAATCTATCGCAAATATTCCTACTAATCAGCTGAAGCTTCGTACGC |
| PSapt-r | AGCCAACTTCAACTCTTGGGCATAACTTGCTATAGACATGCATAGGCCACTAGTGGATC |
| PSaxl-f | GCTTCCAACATCTACGTATATCAAGAAGCATTCACTTACCAGCTGAAGCTTCGTACGC |
| PSaxl-r | AGTAGCTGTCAGCAATAATGAAATCTGAAGCTGTGTCATGCATAGGCCACTAGTGGATC |
| PSade-f | ATTGCTTACAAAGAATACACATACGAAATATTAACGATAAGCTGAAGCTTCGTACGC |
| PSade-r | TGGCAATATACCGTCCAGTTCAGTCTTCGTAATTGACATGCATAGGCCACTAGTGGATC |
| Inserting a promoter (group II) | |
| PCypt-f | CAGGAAATACAAGTAGCACAAAATACGGAGGAATACGATCAGCTGAAGCTTCGTACGC |
| PCypt-r | GAATCTCCTAGCAGTACCACTTTGATGGTTGCTTCCATCTTGGAGTTGATTGTATGCTT |
| PCung-f | ATATATATTATCAGAAGCTGTACACAAGCCGTTCACATACCAGCTGAAGCTTCGTACGC |
| PCung-r | ATAACTGAATTTGTTGGCAATCTTCTCATGCACCACATCTTGGAGTTGATTGTATGCTT |
| PCrev-f | CCAAGAAGAAAAAAAAAATAGTAATCGTTGCGTCAGCTTTCAGCTGAAGCTTCGTACGC |
| PCrev-r | AAGTATACCCTCAGCCACTTCTCTACCCATCTATTCATCTTGGAGTTGATTGTATGCTT |
| PCkin-f | GAGTGCTATTTGATAATTGAATCAATTCTCTGTGTGAGTCAGCTGAAGCTTCGTACGC |
| PCkin-r | GGACTACGGTATTCTTGGAAAAACTGTCGTCTATGCATCTTGGAGTTGATTGTATGCTT |
| Knocking out the downstream gene (group III) | |
| KhgyF | TTAATTAAGGCGCGCCAGATCTGTTTAGCTTGCCTTGTAGCTGAAGCTTCGTACGC |
| KyptR | ATTATTATTTTATTCAATGTATCGAAATTGTACCCTGTCGCATAGGCCACTAGTGGATC |
| KungR | GAGTTTGGAAATCGAGACCTGCATATGCAATAGTAATATGCATAGGCCACTAGTGGATC |
| KrevR | TTAAAACAAAGATCCAAAAATGCTCTCGCCCTCTTCATAGCATAGGCCACTAGTGGATC |
| KkinR | TTCTGGAACCTTACCAACTTTCTTACCGCTAATTACTTCGCATAGGCCACTAGTGGATC |
| Inserting one or two nucleotide bases (group IV) | |
| PCypt-f | CAGGAAATACAAGTAGCACAAAATACGGAGGAATACGATCAGCTGAAGCTTCGTACGC |
| PCypt-r2 ** | GAATCTCCTAGCAGTACCACTTTGATGGTTGCTTCccCATCTTGGAGTTGATTGTATGCTT |
| PCung-f | ATATATATTATCAGAAGCTGTACACAAGCCGTTCACATACCAGCTGAAGCTTCGTACGC |
| PCung-r2 ** | TAACTGAATTTGTTGGCAATCTTCTCATGCACCAgCATCTTGGAGTTGATTGTATGCTT |
| PCrev-f | CCAAGAAGAAAAAAAAAATAGTAATCGTTGCGTCAGCTTTCAGCTGAAGCTTCGTACGC |
| PCrev-r2 ** | AGTATACCCTCAGCCACTTCTCTACCCATCTATTgCATCTTGGAGTTGATTGTATGCTT |
| PCkin-f | GAGTGCTATTTGATAATTGAATCAATTCTCTGTGTGAGTCAGCTGAAGCTTCGTACGC |
| PCkin-r2 ** | GACTACGGTATTCTTGGAAAAACTGTCGTCTATGccCATCTTGGAGTTGATTGTATGCTT |
| Using HO-poly-KanMX4-HO vector (group V) | |
| HO-ypt-f * | tt ggccattacggcc ATGGAAGCAACCATCAAAGTGGT |
| HO-ypt-r * | tt ggccgaggcggcc TGTAATGAATCTAGTGAAGTCG |
| HO-rev-f1 * | tt ggccattacggcc ATGAATAGATGGGTAGAGAAGT |
| HO-rev-r1 * | tt ggccgaggcggcc AGCATGGAACCAGACAGAA |
| HO-ung-f1 * | tt ggccattacggcc ATGTGGTGCATGAGAAGATT |
| HO-ung-r1 * | tt ggccgaggcggcc TGAAAGGCAGGAGTAAGTTG |
| pGU-kinF * | tt ggccattacggcc ATGCATAGACGACAGTTTTTCCAA |
| HO-kin-r * | tt ggccgaggcggcc CTCCTCTAGATTCTGGAACGGTGC |
| Using HO-poly-KanMX4-HO vector (group VI) | |
| Tcyc-f | GAGCAGGACTGACACGTCC |
| HO-cyc-r * | tt ggccgaggcggcc AGCTTGCAAATTAAAGCCTT |
| HO-ypt-f * | tt ggccattacggcc ATGGAAGCAACCATCAAAGTGGT |
| HO-ypt-r o | GGACGTGTCAGTCCTGCTCTTAACATATACAGCCACTGCC |
| HO-rev-f1 * | tt ggccattacggcc ATGAATAGATGGGTAGAGAAGT |
| HO-rev-r o | GGACGTGTCAGTCCTGCTCTTAAAACAAAGATCCAAAAATGC |
| HO-ung-f1 * | tt ggccattacggcc ATGTGGTGCATGAGAAGATT |
| HO-ung-r o | GGACGTGTCAGTCCTGCTCTCAAGGGTCCTTTGATTCTG |
| pGU-kinF * | tt ggccattacggcc ATGCATAGACGACAGTTTTTCCAA |
| HO-kin-r o | GGACGTGTCAGTCCTGCTCTTATCGATATCTTGTTTGCCA |
| Changing the ORF (group VII) | |
| HO-kin-zf | CAATATTTCAAGCTATACCAAGCATACAATCAACTCCAAGATGGCCAAGTTGACCAGTG |
| HO-ypt-zr | CCCACGATAATTACCTGCGTTCCCTGAGCACGGTCCTGTCAGTCCTGCTCCTCGGCC |
| HO-kin-zf | CAATATTTCAAGCTATACCAAGCATACAATCAACTCCAAGATGGCCAAGTTGACCAGTG |
| HO-rev-zr | TTTTCACTAAACTGATGAATAATCAAAGGCCCCACGTCTCAGTCCTGCTCCTCGGCC |
| HO-kin-zf | CAATATTTCAAGCTATACCAAGCATACAATCAACTCCAAGATGGCCAAGTTGACCAGTG |
| HO-ung-zr | AAAATCCTCTACTTGCACTTAATGGAGACGGATGCACTTCAGTCCTGCTCCTCGGCC |
| HO-kin-zf | CAATATTTCAAGCTATACCAAGCATACAATCAACTCCAAGATGGCCAAGTTGACCAGTG |
| HO-kin-zr | ACTTCCCTGGCTCGCTCCTCCACTGCCTGGGTAAATTGTCAGTCCTGCTCCTCGGCC |
| Changing the terminator (group VIII) | |
| PK-Ta-f * | ga agatcttcggctagc GTTGTCGGCTTGTCTACCT |
| PK-Ta-r | AAGGTTTTGGGACGCTCGAAGGCTTTAATTTGCAAGCTGGCCGGTAGAGGTGTGGTCA |
| PK-Z-f | AGCATGAGGTCGCTCTTATTGACCACACCTCTACCGGCCAGCTTGCAAATTAAAGCCTT |
| PK-Z-r * | c gagctc GTTTTAGCCTTAGACATGACTG |
| PK-apt-f * | gc gtcgac AGCAAGTTATGCCCAAGAGT |
| PK-apt-r * | ga agatct TCATTTTTTCAACGCTTCCT |
| PK-ung-f * | cg gaattc GAACTTGAGCAGCAATTTACG |
| PK-ung-r * | c gagctc TCAAGGGTCCTTTGATTCTG |
| PK-ade-f * | ga agatct TTGAGCGAACCAAAGTACAAA |
| PK-ade-r * | cg gctagc TTAGTGAGACCATTTAGACCC |
| PK-kin-f * | cg gaattc tt gcggccgc TGCCACAACATACGTCGGTAC |
| PK-kin-r * | c gagctc TTATCGATATCTTGTTTGCCA |
| Group IX & X were constructed using the same primers used to construct group I and II | |
| **Creating tandem strains through a modified T-vector** | |
| U ko kin-f1 | AAGTTCACATCTATCATTGAATGTATTATGGCATTAAG TAATGTGGCTGTGGTTTCAGG |
| U ko kin-r1 | GCTTTTTTTCTTTTCTTTTTTTTTTCAATTGGGGATTA ACTTGGTTCTGGCGAGGTATT |
| KIN-L-f * | at gggccc CGTGATTTACATATACTACAAGTCG |
| KIN-L-r * | TAAATGTATGAACGACTCAAAGTGCATgacgtcatCTTAATGCCATAATACATTCAATG |
| KIN-R-f * | at gacgtc ATGCACTTTGAGTCGTTCATAC |
| KIN-R-r * | tt gcatgc GTAGTTCTCCAACAAAAGATGAGC |
| Gap-kin-f2 * | at gacgtc GGGATTAAATGTATGAACGACTC |
| Gap-kin-r3 * | at caattg TTAAGAGACTGGCTTACTGCTA |
| **Dual luciferase assay** | |
| Replacing the target regions by the reporter *Fluc* | |
| PK-Fluc-f * | ga agatct ATGGAAGACGCCAAAAACAT |
| PK-Fluc-r * | c gagctc TTACACGGCGATCTTTCCG |
| Fluc-apt-5f * | gc gtcgac TGGCACTCCAGAAACAACA |
| Fluc-apt-5r * | ga agatct GATTAGTAGGAATATTTGCGATAG |
| Fluc-apt-3f * | c gagctc TGAAAGGCAGGAGTAAGTTG |
| Fluc-apt-3r2 * | cg gaattc ATATTACTATTGCATATGCAGGT |
| Fluc-ade-5f * | gc gtcgac AACACGTCCAACTCCTTAA |
| Fluc-ade-5r * | ga agatct TATCGTTAATATTTCGTATGTGT |
| Fluc-ade-3f * | c gagctc TCCTCTAGATTCTGGAACGG |
| Fluc-ade-3r * | cg gaattc GCCAAACCTGCATACCACT |
| URA-kAPT-f | GCTTCAAAGAAATCTATCGCAAATATTCCTACTAATC ATGTCGAAAGCTACATATAAGG |
| URA-kAPT-r | AGTGAACACTGGAGCATTCAACTTACTCCTGCCTTTCAACTTGGTTCTGGGGAGGTATT |
| URA-kADE-f | TGCTTACAAAGAATACACATACGAAATATTAACGATA ATGTCGAAAGCTACATATAAGG |
| URA-kADE-r | CCTACCTTATAAGAGGCACCGTTCCAGAATCTAGAGGAACTTGGTTCTGGGGAGGTATT |
| Inserting the internal control *Rluc* | |
| p15-f * | gc gtcgac tcg gctagc AACAGCAGAAACTCGCAACG |
| p15-r | TCCTTTGTTCTGGATCATAAACTTTCGAAGTCATAATTAAAAAAGTTTCTCTTGATACA |
| PK-Rluc-f | CATAAACAGGTGTATCAAGAGAAACTTTTTTAATTATGACTTCGAAAGTTTATGATCC |
| PK-Rluc-r | TTCGTAAATTTCTGGCAAGGTAGACAAGCCGACAACTTATTGTTCATTTTTGAGAACTC |
| Ta-f | TCGTTCGTTGAGCGAGTTCTCAAAAATGAACAATAAGTTGTCGGCTTGTCTACCT |
| Ta-r * | c gagctc GGCCGGTAGAGGTGTGGTCA |
| OI-U5-f * | gc gtcgac TAATGTGGCTGTGGTTTCAGG |
| OI-U5-r * | tcg gctagc GGTTAAGAATACTGGGCAAT |
| OI-U3-f * | c gagctc CCGTGGATGATGTGGTCTCT |
| OI-U3-r * | cg gaattc ACTTGGTTCTGGGGAGGTATT |
| **Cell-cycle synchronization** (knocking out *BAR1*) | |
| Kbar-f | AGTGGTTCGTATCGCCTAAAATCATACCAAAATAAAAAGAGCTGAAGCTTCGTACGC |
| Kbar-r | GATATTTATATGCTATAAAGAAATTGTACTCCAGATTTCGCATAGGCCACTAGTGGATC |
| **Nascent RNA expression analysis** | |
| ADE1-nascent-RT1 | CACTGGCAAACAAGATATCG |
| APT1-nascent-RT1 | ATATTACTATTGCATATGCAGGTC |
| KIN3-nascent-RT1 | AGAGACTGGCTTACTGCTAATAAG |
| UNG1-nascent-RT1 | AAATGATATGTTTCACGTCCTG |
| **Profiling gene expression in single cells** | |
| nest-KIN3-f | GCCACAACATACGTCGGTACA |
| nest-KIN3-r | AGGATTTTTTCAATGTTTGTCAGC |
| nest-ADE1-f | TCTTCACCCCATCGACCAA |
| nest- ADE1-r | CAGTAAGCCAGTCTCTTAAAAATTGC |
| nest-ACT1-f | GCACAGAGCCTCGCCTTT |
| nest-ACT1-r | CGTGCTCGATGGGGTACTTC |
| RT-nest-ACT1 | GTTGTCGACGACGAGCG |

** The lowercase in the primers means the nucleotide bases for making frame shift mutagenesis.
